# Shen-Bai-Jie-Du decoction suppresses the progression of colorectal adenoma to carcinoma through regulating gut microbiota and short-chain fatty acids

**DOI:** 10.1186/s13020-024-01019-4

**Published:** 2024-10-28

**Authors:** Min Huang, Ye Zhang, Mingxin Ni, Meng Shen, Yuquan Tao, Weixing Shen, Dongdong Sun, Liu Li, Changliang Xu, Jiani Tan, Yueyang Lai, Chengtao Yu, Lihuiping Tao, Minmin Fan, Haibo Cheng

**Affiliations:** 1 The First Clinical Medical College, Jiangsu Collaborative Innovation Center of Traditional Chinese Medicine Prevention and Treatment of Tumor, Nanjing University of Chinese Medicine, Nanjing, 210023 China; 2 Department of Oncology, Jiangsu Province Hospital of Chinese Medicine, Affiliated Hospital of Nanjing University of Chinese Medicine, Nanjing, 210029 China; 3 The First Affiliated Hospital of Soochow University, Soochow, 215123 China

**Keywords:** Shen-Bai-Jie Du decoction, Short-chain fatty acids, Colorectal adenoma, Gut microbiota

## Abstract

**Background:**

Shen-Bai-Jie-Du decoction (SBJDD), a traditional Chinese herb formula developed based on evidence-based medicine, is efficacy to reduce the recurrence and carcinogenesis of colorectal adenoma. However, the mechanism of SBJDD to treat colorectal adenoma remains unclear. The present study aims to investigate the efficacy and mechanism of SBJDD on colorectal adenoma carcinogenesis from the aspects of regulating gut microbiota and short-chain fatty acids (SCFAs).

**Methods:**

Twenty-one patients diagnosed with colorectal adenoma were recruited in the study and required to take SBJDD for four consecutive weeks. Analysis of gut microbiota was conducted using 16S rRNA gene amplicon sequencing, while levels of SCFAs in fecal and serum samples were determined through HPLC–MS/MS. Additionally, twenty-four Apc^min/+^ mice were randomly assigned to normal diet (ND), high-fat diet (HFD), and SBJDD groups. The pharmacological effects and mechanism of SBJDD on colorectal adenoma carcinogenesis were assessed using RT-qPCR, HE staining, IHC staining, Western blot, IF staining, and Flow cytometry assays.

**Results:**

Our clinical study has shown that SBJDD can regulate the gut microbiota composition and enhance SCFAs production in patients with colorectal adenoma. SBJDD alleviated colorectal adenoma formation and carcinogenesis, as well as protected the integrity of the intestinal barrier in the Apc^min/+^ mice model compared to the HFD group. Additionally, SBJDD was found to regulate gut microbiota capable of producing SCFAs. G protein-coupled receptors GPR43, GPR41, and GPR109a were effectively activated in the SBJDD group, while HDAC1 and HDAC3 were inhibited. Furthermore, decreased expression levels of interleukin 1 beta (IL-1β) and interleukin 6 (IL-6), along with elevated expression level of interleukin 10 (IL-10), were observed in the colorectal tissue of the SBJDD group. Finally, SBJDD exhibited the ability to reduce the proportion of M1-type macrophages while increasing the proportion of M2-type macrophages.

**Conclusions:**

Our study objectively demonstrated the pharmacological effects of SBJDD in inhibiting the progression of colorectal adenoma and investigated its mechanisms in terms of regulating gut microbiota, increasing SCFAs, and reducing colorectal inflammation.

**Supplementary Information:**

The online version contains supplementary material available at 10.1186/s13020-024-01019-4.

## Introduction

Colorectal cancer (CRC) is one of the most commonly diagnosed malignancies worldwide with limited treatment options [1]. Approximately 85% of CRC are thought to evolve from traditional pre-cancerous colorectal adenomas, a process known as the adenoma to cancer sequence [2]. Despite the increasing use of colonoscopy screening leading to more frequent diagnosis and endoscopic removal of colorectal adenomas, about 30% of patients remain at risk for recurrence after polypectomy [3]. Therefore, the prevention of colorectal adenoma recurrence and carcinogenesis remains a great challenge. At present, there is no recognized effective treatment strategies for preventing colorectal adenoma recurrence and carcinogenesis at home and abroad.

Traditional Chinese medicine (TCM) prescriptions have been used to prevent and treat CRC under the guidance of TCM theory for thousands of years [4]. However, the biological basis of most TCM is yet to be determined. Emerging evidence suggests that TCM may exert their beneficial effects through modulation of gut microbiota and metabolites [5]. Therefore, it is crucial to offer new perspectives on the pharmacological properties of TCM in the prevention and treatment of CRC.

Shen-Bai-Jie-Du decoction (SBJDD), a traditional Chinese herb formula developed based on the theory of cancer pathogenesis by professor Haibo Cheng. This prescription is composed of Hedyotis Diffusa, Sophorae Flavescentis Radix, Codonopsis Radix, Atractylodis Macrocephalae Rhizoma, Coicis Semen, Coptidis Rhizoma, Zingiberis Rhizoma Praeparatum and Mume Fructus. The combination of various herbs in the prescription plays the role of “anti-cancer and detoxification, clearing heat and drying dampness, strengthening spleen and supplementing qi”. We have recently completed a 400-patient, multicenter, randomized, double-blind, placebo-controlled clinical trial to evaluate efficacy of SBJDD by eight hospitals in China. Our results demonstrated that SBJDD significantly reduced the recurrence rate of colorectal adenoma by 16.07% while maintaining a high level of safety [6]. As we all know, the recurrence of colorectal adenoma is closely related to the carcinogenesis [7]. However, the mechanism by which SBJDD inhibits adenoma carcinogenesis remains poorly understood and requires further investigation.

Short-chain fatty acids (SCFAs) are the major microbial fermentation products and are presumed to have a crucial role in host physiology. When dietary fiber is not sufficient, it will lead to a reduction in the activity of the fermenting microbiota involved and SCFAs as minor end products. SCFAs including acetate, propionate, butyrate, etc., can act on their cognate receptors such as GPR41 and GPR43 to perform multiple functions. They can also serve as histone acetylation inhibitors, interleukin 6 inhibitors, and STAT3/IL-17 pathway activators to exert anti-tumor and anti-inflammatory effects [8, 9]. In addition, SCFAs can down-regulate the Wnt signaling pathway and inhibit the proliferation and migration of cancer cells [10]. In this study, we revealed that SBJDD can regulate gut microbiota, promote the production of SCFAs, protect the intestinal barrier and alleviate colorectal inflammation. Finally, our results suggest SBJDD as a potential chemopreventive agent in colorectal adenoma to carcinoma progression and underlie its underlying molecular mechanisms in inhibiting colorectal adenoma formation and carcinogenesis.

## Materials and methods

### Clinical sample collection

Twenty-one patients with colorectal adenoma were recruited from the Gastroenterology Endoscopy Center at Jiangsu Province Hospital of Chinese Medicine. This study was approved by the Ethics Committee of Jiangsu Provincial Hospital of Chinese Medicine (Ethic code: 2018NL-067–02).

The inclusion criteria are as follows: (1) Patients are pathologically confirmed conventional colorectal adenoma during colonoscopy; (2) Patients range in age from 18 to 75; (3) Patients have not undergone polypectomy; and (4) Patients have signed informed consent according to the requirements of the GCP (Good Clinical Practice).

The exclusion criteria are as follows: (1) Patients with Familial adenomatous polyposis, Mutyh-associated polyposis, Peutz-Jeghers syndrome and other hereditary polyposis; (2) Patients with a history of colorectal cancer, inflammatory bowel disease or irritable bowel syndrome; (3) Patients taking antibiotics or probiotics within 2 weeks; (4) Patients with serious diseases of the heart, liver and kidney; (5) Patients with autoimmune diseases, chronic wasting diseases, brain metastases or mental disorders, pregnant and nursing women; and (6) Poor compliance or participation in other clinical trials.

All patients were enrolled in the study and required to take SBJDD at the dose of 1.57 g/kg per day for four consecutive weeks. Fecal samples and serum samples were collected before and after treatment. The basic information and pathological conditions of patients enrolled in the study are shown in Supplementary Table 1.

### 16S rRNA gene sequence analysis

Fresh fecal samples from participants were snap-frozen and stored at − 80 ℃ after collection, Bacterial DNA was isolated from the fecal samples using a MagPure Soil DNA LQ Kit (Magen, Guangdong, China) following the manufacturer’s instructions. DNA concentration and integrity were measured by a NanoDrop 2000 spectrophotometer (Thermo Fisher Scientific, Waltham, MA, USA) and agarose gel Electrophoresis, respectively. The PCR products were purified with Agencourt AMPure XP beads (Beckman Coulter Co., USA) and quantified using a Qubit dsDNA assay kit. The concentrations were adjusted for sequencing. Sequencing was performed on an Illumina NovaSeq6000 with two paired-end read cycles of 250 bases each (Illumina Inc., San Diego, CA; OE Biotech Company; Shanghai, China). Paired-end reads were preprocessed using Trimmomatic software to detect and cut off ambiguous bases (N). It also cut off low-quality sequences with an average quality score below 20 using a sliding window trimming approach. After trimming, paired-end reads were assembled using FLASH software. The parameters of assembly were: 10 bp of minimal overlapping, 200 bp of maximum overlapping, and 20% of maximum mismatch rate. Sequences were performed further denoising as follows: reads with ambiguous, homologous sequences or below 200 bp were abandoned. Reads with 75% of bases above Q20 were retained using QIIME software (version 1.8.0). Then, reads with chimera were detected and removed using VSEARCH. Clean reads were subjected to primer sequences removal and clustering to generate operational taxonomic units (OTUs) using VSEARCH software with 97% similarity cutoff. α-Diversity was calculated based on the Chao1 Shannon and Simpson diversity index.

Meta-statistics analysis was calculated based on linear discriminant analysis coupled with effect size measurements and Wilcoxon analysis of variance. The 16S rRNA gene amplicon sequencing and analysis were conducted by OE Biotech Co., Ltd. (Shanghai, China).

### Preparation of SBJDD

The eight kinds of herbs used in SBJDD were purchased from Yabang Chinese Herbal Beverage Company in Jiangsu, China. All herbs were authenticated by Professor Si-li Zou (Nanjing University of Chinese Medicine). 20 g Hedyotis Diffusa, 9 g Sophorae Flavescentis Radix, 15 g Codonopsis Radix, 12 g Atractylodis Macrocephalae Rhizoma, 20 g Coicis Semen, 3 g Coptidis Rhizoma, 6 g Zingiberis Rhizoma Praeparatum and 9 g Mume Fructus were immersed in water (1:10, w/v) for one hour and then heated to boiling and hold for two hours. The aqueous extract was filtered and the herb residue was subjected to decocted again in water (1:8, w/v) under the same conditions. The filtrate was combined and precipitated with 60% alcohol for one day. A rotary evaporator was used to concentrate the solution to a concentration of 2 g per milliliter.

### Reagents and antibodies

Co60-irradiated purified processed feed (60% fat) (no. XTHF60) and maintenance feed (no. SWS9102) for experimental animals were purchased from Synergy Pharmaceutical & Biological Engineering Co. (Jiangsu, China). Anti-Ki67 (no. sc-23900), anti-β-catenin (no. sc-7963), anti-ZO-1 (no. sc-33725), anti-Occludin (no. sc-133256), anti-Claudin-1 (no. sc-81796), anti-F4/80 (no. sc-52664) and anti-GPR109a (HM74, no. sc-377292) antibodies were purchased from Santa Cruz Biotechnology (Santa Cruz, USA). Anti-GPR41 (FFAR3, no. 66811–1-Ig), anti-GPR43 (FFAR2, no. 19952–1-AP) were purchased from Proteintech Group (Chicago, IL, USA). Anti-GAPDH (no. 2118S), anti-β-Tubulin (no. 2146S) and Mouse Reactive M1 vs M2 Macrophage IHC Antibody Sampler Kit (no. 97624 T) were obtained from Cell Signaling Technology (Beverly, USA). Alexa Fluor™ 594 goat anti-rabbit lgG (H + L) antibody (no. 35560), AlexaFlour™ 488 donkey anti-mouse antibody (no. R37114), F4/80 monoclonal antibody (PE-Cyanine7) (no. 25–4801-82), CD11b monoclonal antibody (FITC) (no. 11–0112-82), CD45 monoclonal antibody (PerCP-Cyanine5.5) (no. 45–0451-82), CD86 monoclonal antibody (APC) (no. 17–0862-82) and CD206 monoclonal antibody (PE) (no. 12–2061-82) were purchased from Invitrogen (Massachusetts, USA). GTVisionTM III Detection System/Mo&Rb (Including DAB) was purchased from Gene Tech (Shanghai, China), and DAPI (no. C1002) was from Beyotime Biotechnology (Shanghai, China).

### Identification and analysis of components in SBJDD by UPLC-MS/MS

**Fig. 1:**
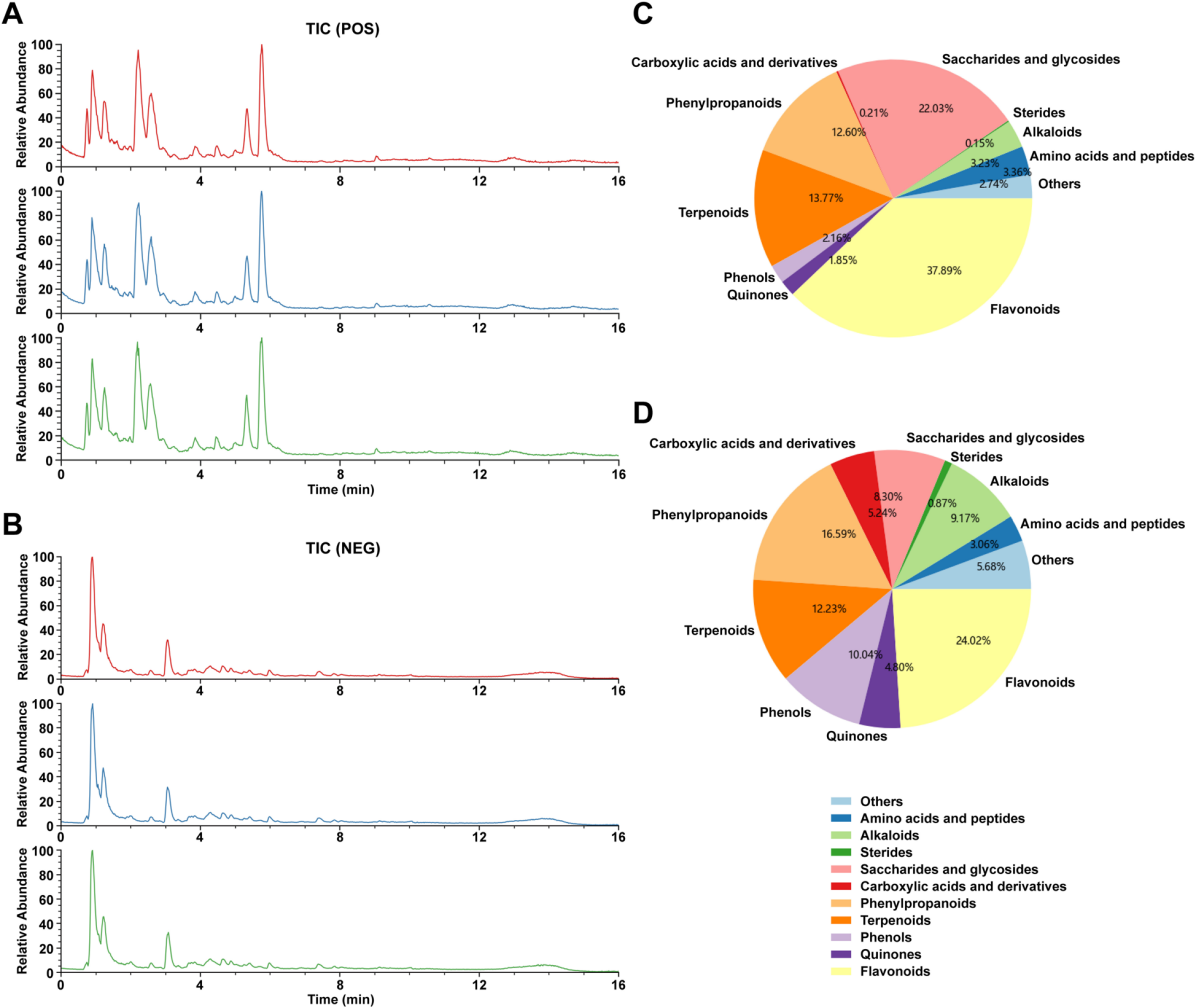
The UPLC-MS/MS analysis results of SBJDD. **A** Total ion flow chart of sample in the positive ion mode (N = 3). **B** Total ion flow chart of sample in the negative ion mode (N = 3). **C** The proportion of each component content in SBJDD. **D** The proportion of each component classification in SBJDD

UPLC-MS/MS analysis was made using a very efficient liquid chromatograph ACQUITY UPLC I-Class plus (Waters, Milford, USA) coupled with a mass spectrometer Thermo-Obritrap-QE (Waters, Milford, MA, USA). Chromatographic separation was performed on ACQUITY UPLC HSS T3 Column (100 mm × 2.1 mm, 1.8 μm) (Waters, Ireland) at 45 ℃ with 0.35 mL/min flow rate. The injection volume of the sample was set to 5 μL. The mobile phase consisted of 0.1% formic acid in water (A) and acetonitrile (B). Multistep gradient elution of the mobile phase was programed as follows:0-2 min, 5–5% B; 2–4 min, 5–30% B; 4–8 min, 30–50% B; 8–10 min, 50–80% B; 10–14 min, 80–100% B; 14–15 min, 100% B; 15–15.1 min, 100–5% B; 15.1–16 min, 5% B; Ionization mode: Electro-Spray Ionization (ESI). Both positive and negative ion mass spectral signals were acquired. The positive ion mode was as follows: the spray voltage was 3800 V, the capillary temperature was 320℃, the aux gas heater temperature was 350 ℃, the sheath gas flow rate was 35 Arb, the aux gas flow rate was 8 Arb, the S-lens RF level was 50, the mass range (m/z) was from 100 to 1200, the full MS resolution was 70,000, the MS/MS resolution was 17,500, the NCE/stepped NCE was 10, 20, 40; The negative ion mode is as follows: the spray voltage was − 3000 V, the capillary temperature was 320 ℃, the aux gas heater temperature was 350 ℃, the sheath gas flow rate was 35 Arb, the aux gas flow rate was 8 Arb, the S-lens RF level was 50, the mass range (m/z) was from 100 to 1200, the full MS resolution was 70,000, the MS/MS resolution was 17,500, the NCE/stepped NCE was 10, 20, 40; The raw data were preprocessed by metabolomics processing software Progenesis QI v3.0 (Nonlinear Dynamics, Newcastle, UK). Compounds were identified based on precise mass numbers, secondary fragments, and isotopic distributions and characterized using the TCM database (lumingbio, Shanghai, China). The UPLC-MS/MS analysis results and detailed compounds information for SBJDD are shown in Fig. 1 and Supplementary Table 2.

### Mice and establishment of model

The experiments were conducted under the supervision and guidance of the Experimental Animal Ethics Committee of Nanjing University of Chinese Medicine, in accordance with the requirements of experimental animal ethics (Ethic code: 202109A041). The C57BL/6J Apc^min/+^ mouse model is a well-established animal model for investigating the pathogenesis of intestinal tumorigenesis, characterized by spontaneous formation of colorectal adenomas. This model has been extensively utilized in research pertaining to the carcinogenesis of colorectal adenoma [11, 12]. At 4 weeks of age, Apc^min/+^ mice (strain NO. T001457) were procured from Jiangsu GemPharmatech (Jiangsu, China) and housed in the Experimental Animal Centre of Nanjing University of Chinese Medicine. The housing conditions included a relative humidity of 55 ± 15%, an ambient temperature of 23 ± 3 ℃, and a 12-h light/dark cycle, with ad libitum access to food and water.

Multiple studies have shown that high-fat diet (HFD) may induce or exacerbate intestinal inflammation and chronic inflammation is a recognized risk factor for colorectal carcinogenesis [13]. After one week of the adaptation period, twenty-four mice were randomly allocated into three groups (N = 8). The three groups are ND group (continuous maintenance feed and intragastric administration of saline), HFD group (continuous 60% fat purified processed feed and intragastric administration of saline), and SBJDD group (continuous 60% fat purified processed feed and intragastric administration of SBJDD). In our clinical trial, patients were administered a dosage of 94 g per day of SBJDD [6], which corresponds to 1.5667 g/kg based on the average body weight of 60 kg. Considering the conversion coefficient between mice and human as 9.1, the dosage for mice was calculated as 1.5667 g/kg × 9.1 = 14.257 g/kg. Therefore, the intragastric administration of SBJDD in mice was performed at a dose of approximately 14.3 g/kg per day, equivalent to the human equivalent dose. The SBJDD and saline were administered six consecutive days in one week. The body weight of mice was measured every two days. After 12 weeks, the mice were sacrificed, the colorectal tissue of the mice was dissected along the longitudinal axis, and feces were immediately collected. The number, size, and location of adenomas were recorded. Part of the colorectal tissue was fixed in 4% paraformaldehyde and then made into paraffin-embedded tissue sections for HE staining or immunohistochemical staining. The remaining part of the colorectal tissue was stored at − 80 ℃ for subsequent analysis.

### Determination of fecal gut microbiota abundance in mice

Fresh fecal samples of mice in each group were collected and put into sterile collection tubes. The genomic DNA of fecal samples of mice was extracted using TIANamp Stool DNA Kit (no. DP328, Tiangen Biochemical Technology Co., LTD., Beijing, China). Real-time quantitative PCR analysis was used to detect the relative abundance of specific strains in fecal samples and perform statistical analysis. The abundance levels of all specific strains were based on the abundance of 16S rRNA. The primer sequences used for amplification are shown in Supplementary Table 3.

### Short-chain fatty acids (SCFAs) analysis

Multiple reaction monitoring (MRM) was used to make quantification of SCFAs, including butyrate, isobutyrate, 2-methyl-butyrate, propionate, acetate, valerate, and Isovalerate. In a nutshell, 20 mg of homogenized fecal samples were weighed and dissolved in 1 mL of 70% acetonitrile. The sample was vortexed until completely mixed and then centrifuged at 10 ℃ and 4000*g* for 10 min. 40 μL of supernatant was taken to be derivatized, 5 μL of d3-hexanoic acid (10 μg/mL) was added to the supernatant, vortexed for 3 min, then 20 μL of 200 mM 3-NPH and 20 μL of 120 mM EDC-HCl-6% pyridine solution were added, vortexed for 3 min, and the reaction was carried out at 40 ℃ for 30 min, then centrifuged at 18,000 rpm for 10 min. The supernatant was taken into in a 2 mL screw-cap glass vial, and then subjected to SCFAs analysis with high-performance liquid chromatography coupled with a triple-quadruple mass tandem spectrometer (HPLC–MS/MS) (Thermo Fisher Scientific, USA).

### Real-time quantitative PCR analysis

RNAiso Plus reagent (Takara, Japan) was used for Total RNA extraction of colon tissues and 1 µg of RNA was reverse transcribed into 20 μL of complementary DNA using 5 × RT buffer (TOYOBO, Japan), Real-time quantitative PCR analysis was performed using 2 × ChamQ Universal SYBR qPCR Master Mix (Vazyme, Nanjing, China) on StepOnePlus Real-Time PCR System (Thermo Fisher Scientific, USA), The specific reagent ratios and experimental conditions were consistent with previously described [14]. The primer sequences used for amplification are shown in Supplementary Table 3, the transcript levels of all target genes were normalized to *β*-actin mRNA expression.

### Western blot

Western blot analysis was performed as previously described [15]. The densitometry of immunoblots was quantified with Image J software.

### Hematoxylin and eosin (HE) staining

The colorectal tissue was dissected longitudinally and washed with PBS, then the colorectal tissue was fixed by needle after rolling. The rolled colorectal tissue was routinely dehydrated, buried and sectioned at 4 µm thickness using HE staining. Using Nikon ECLIPSE TS100 microscope (Tokyo, Japan) to observe and histological images were taken for documentation.

### Immunohistochemistry (IHC) assay

The colorectal tissue sections were immersed in sodium citrate antigen repair reagent (Beyotime Biotechnology, Shanghai, China), heated to boiling, and held for 15 min for antigen retrieval in the microwave oven. After cooling to room temperature, the sodium citrate buffer was poured off. The sections were washed in double-distilled water and treated with 2% H_2_O_2_ to inactivate endogenous peroxidase activity. Then using 0.2% Triton X-100 to permeable tissue. The colorectal tissue sections were washed 3 times with PBS and then blocked with 3% normal goat serum (Beyotime Biotechnology, Shanghai, China) for one hour. Primary antibodies diluted 1:200 were incubated overnight at 4℃ and secondary antibodies were incubated at room temperature for 30 min. The sections were followed by treatment with DAB solution for 10 min and stained with hematoxylin staining solution for nuclear staining. Images were captured using Nikon ECLIPSE TS100 microscope (Tokyo, Japan). The IHC score was calculated using Image J software.

### Immunofluorescent (IF) staining

The colorectal tissue sections were immersed in sodium citrate antigen repair reagent (Beyotime Biotechnology, Shanghai, China), heated to boiling, and held for 15 min for antigen retrieval in the microwave oven. After cooling to room temperature, the sodium citrate buffer was poured off. The sections were washed in double-distilled water and treated with 2% H_2_O_2_ to inactivate endogenous peroxidase activity. Then using 0.2% Triton X-100 to permeable tissue. The colorectal tissue sections were washed 3 times with PBS and then blocked with 3% normal goat serum (Beyotime Biotechnology, Shanghai, China) for one hour. Primary antibodies diluted 1:200 were incubated overnight at 4℃ and fluorescent secondary antibodies were incubated at room temperature in dark condition for 2 h. The sections were stained with DAPI for nuclear staining. Images were captured using Leica Laser Scanning Confocal Microscopy TCS SP8 (Germany).

### Flow cytometry

A total of 5 mL of pre-cooled PBS was injected into the abdominal cavity of the mice to obtain the peritoneal lavage fluid, and the red blood cells were removed using red blood cell lysate and the cells were collected by centrifugation. The cells were resuspended with 100 μL stain buffer, then 0.5 μL FcR blocking was added and incubated on ice for 10 min away from light. Then the suspension cells were added with 900 μL stain buffer and centrifuged at 400 g for 5 min. The supernatant was discarded and the cells were resuspended with 100 μL stain buffer. The CD45, F4/80, CD11b and CD86 antibodies were added to the stain buffer, the cells were incubated on ice for 30 min, then 1 mL stain buffer was added to wash the cells immediately, the cell fixation buffer was used to fix the cells. Then the cells were added with the membrane breaking solution, centrifuged at 400*g* for 5 min, then resuspended with 100 μL membrane breaking solution, and incubated with CD206 antibody for 30 min in dark light. Finally, the cells were washed with 1 mL of membrane breaking solution, the cells were resuspended by adding 500 μL stain buffer and filtered by 70 μm nylon mesh, then detected by flow cytometry. The flowjo software was used to analyze the proportion of M1 and M2 macrophages and the mean fluorescence intensity of CD86 and CD206.

### Statistical analysis

The GraphPad Prism (version 8) was used to conduct statistical analysis, T-test was used to conduct the comparison between the two groups of data, and one-way ANOVA was used for the comparison of data from more than two groups. The categorical data were compared using the χ^2^ test. In Results are represented as mean ± SEM and *P* < 0.05 was considered statistically significant.

## Results

### SBJDD altered the composition of gut microbiota and production of SCFAs in patients with colorectal adenoma

**Fig. 2:**
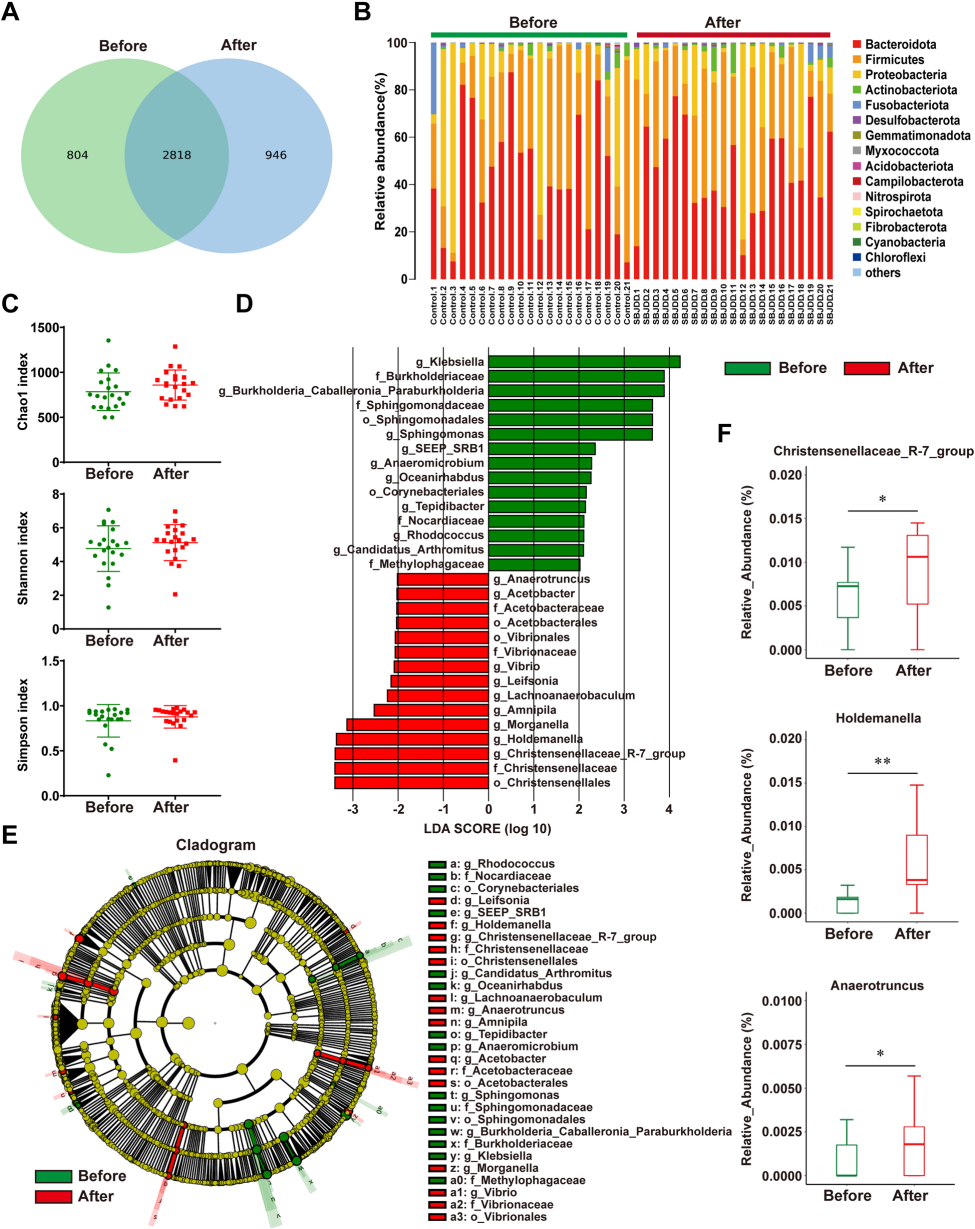
Effects of SBJDD administration on the fecal microbiota in 21 patients with colorectal adenoma. **A** Venn diagram of OTUs cluster analysis results, the green section represents the OTUs before taking SBJDD, the blue section represents OTUs after taking SBJDD, the overlap represents shared OTUs. **B** Relative abundance under phylum classification level of dominant (top 15 and others) gut microbial are represented for each patient before (green, N = 21) and after (red, N = 21) taking SBJDD. **C** Alpha diversity indices (Chao 1 index, Shannon index, Simpson index) of microbiota based on 16SrRNA sequencing in the fecal of patients before (green, N = 21) and after (red, N = 21) taking SBJDD. **D**, **E** Histogram and cladogram of the LDA score based on linear discriminant analysis coupled with effect size measurements showing the enriched bacteria and taxa. **F** Box diagrams of relative abundance of three genera including Christensenellaceae_R − 7_group, Holdemanella and Anaerotruncus. Willcoxon algorithm was used. p-values are indicated on each graph. *P < 0.05, **P < 0.01

**Fig. 3:**
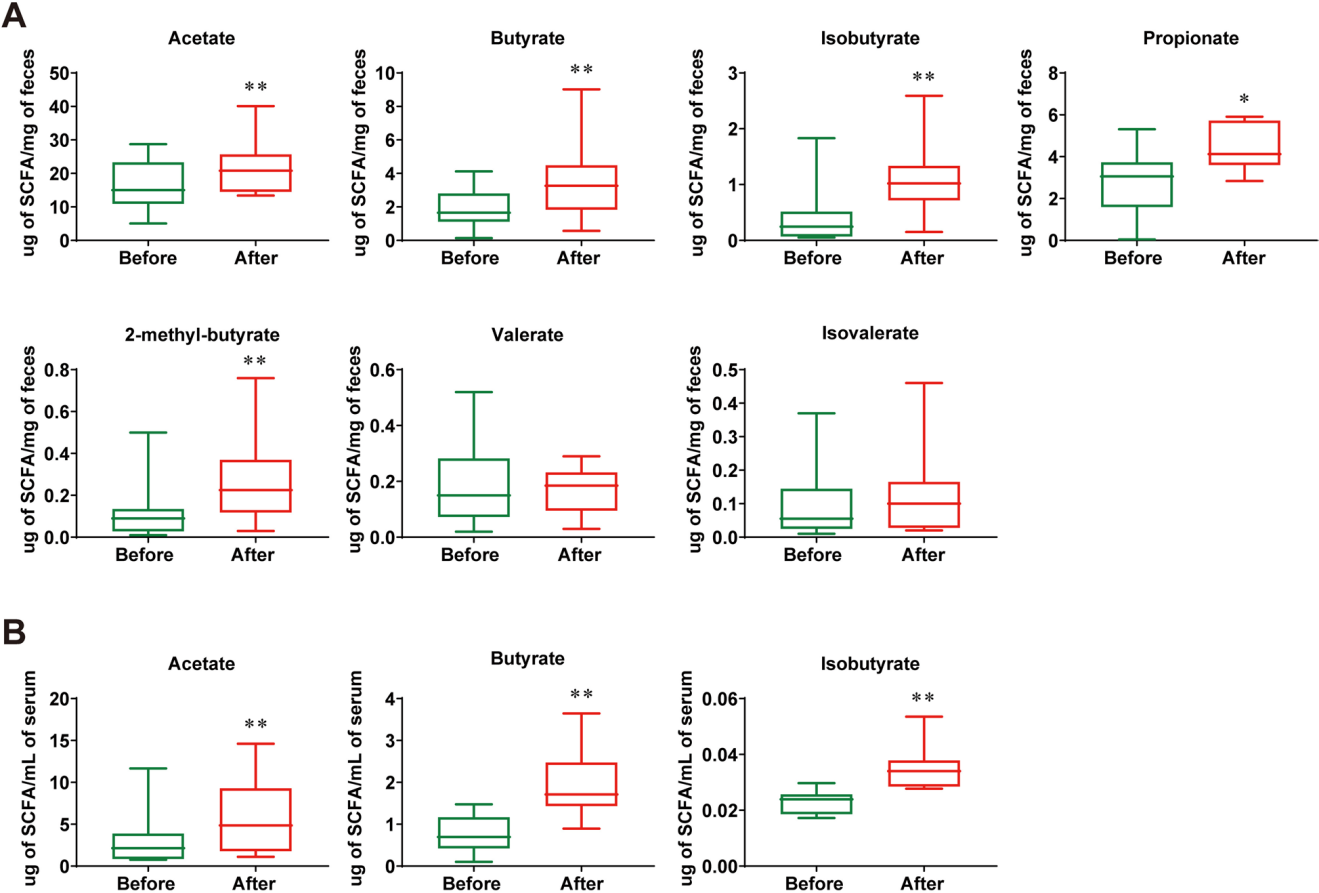
Noticeable changes in the short chain fatty acids (SCFAs) of fecal and serum samples in colorectal adenoma patients. **A** The concentrations of acetate, butyrate, isobutyrate, propionate, 2-methyl-butyrate, valerate and isovalerate in fecal samples of patients before (green, N = 10) and after (red, N = 10) taking SBJDD were determined by high performance liquid chromatography coupled with a triple-quadruple mass tandem spectrometer (HPLC–MS/MS). **B** The concentrations of acetate, butyrate, isobutyrate in serum samples of patients before (green, N = 10) and after (red, N = 10) taking SBJDD were determined by HPLC–MS/MS. *P < 0.05, **P < 0.01

Fecal and serum samples from a total of 21 patients with colorectal adenoma were collected to investigate whether SBJDD could modulate the gut microbiota composition. The Venn graph demonstrated a total of 2818 operational taxonomic units (OTUs) shared in fecal samples from patients before and after taking SBJDD, with 804 OTUs specific to pre-dosing and 946 OTUs specific to post-dosing (Fig. 2A). Relative abundance under phylum classification level of dominant gut microbial analysis result showed that the top 15 were Bacteroidetes, Firmicutes, Proteobacteria, Actinobacteria, Fusobacteriota, Desulfobacterota, Gemmatimonadota, Myxococcota, Acidobacteria, Campilobacterota, Nitrospirota, Spirochaetota, Fibrobacterota, Fibrobacterota and Chloroflexi (Fig. 2B). Meanwhile, we compared the differences in the three indices representing alpha diversity between the two sample groups which were the Chao1index, Shannon index, and Simpson index (Fig. 2C), we found an upward trend in all three indices in the post-drug sample but unfortunately no significant difference. Subsequently, the LEfSe analysis method in statistical analysis of microbial multivariate variables was performed to count the species with significant differences between groups (Fig. 2D, E), from which we found out the order Christensenellales, the family Christensenellaceae, and the genus Christensenellaceae R-7 group exhibited comparative enrichment in the samples after SBJDD administration, Christensenellaceae is a family of firmicute and Le Gall et al. had shown that healthy people have elevated levels of Christensenellaceae compared to CRC patients [16]. The increase in Christensenellaceae R-7 group was also associated with metabolic improvements [17, 18]. Genus level-based Wilcoxon algorithm analysis revealed a significant increase in the genus Christensenellaceae R-7 group, and genus Holdemanella, genus Anaerotruncus in the post-dose samples (Fig. 2F), all of which belong to the Firmicutes. Thus far, some studies have indicated that Firmicutes can metabolize dietary plant-derived polysaccharides to SCFAs and thereby contribute to homeostasis [19, 20]. In the next step, we assessed SCFAs levels in fecal samples and serum samples from a total of 10 patients before and after taking SBJDD. As shown in Fig. 3A, a notifiable increase in fecal SCFAs (acetate, butyrate, isobutyrate, and 2-methyl-butyrate: *P* < 0.01; propionate: *P* < 0.05) levels after SBJDD administration. As some SCFAs are too low in the patients' serum to be detected accurately, we analyzed the three detectable SCFAs and found that the levels of acetate, butyrate, and isobutyrate increased after SBJDD administration (Fig. 3B, P < 0.01).

### SBJDD alleviated colorectal adenoma carcinogenesis and protected the intestinal barrier function in the Apc^min/+^ mice model

**Fig. 4:**
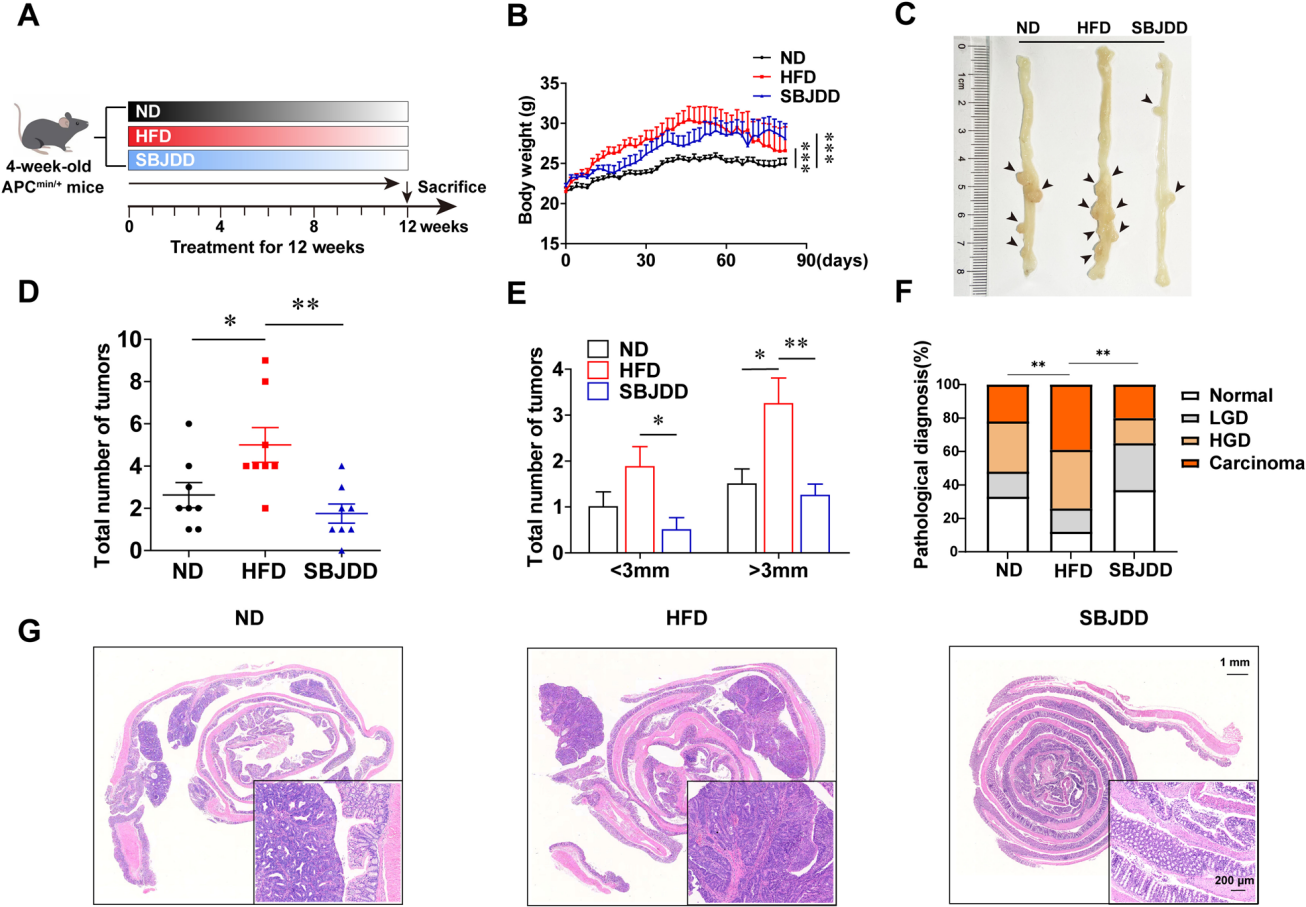
Effects of HFD and SBJDD administration on spontaneously form colorectal adenomas model of Apc^min/+^ mice. **A** Schematic of in vivo experimental procedure (ND: normal diet group, HFD: high-fat diet group, SBJDD: Shen-Bai-Jie-Du decoction group). **B** Body weight change. **C** Representative colorectal appearances. **D** Statistical graph of a total number of tumors. ND (black, N = 8), HFD (red, N = 8) and SBJDD (blue, N = 8). **E** Statistical graph of a total number of tumors diameter larger than 3 mm and smaller than 3 mm. **F** The quantitative analysis of the pathologic score was determined based on the following criteria: 0 for normal, 1 for low-grade dysplasia (LGD), 2 for high-grade dysplasia (HGD), and 3 for carcinoma. **G** Representative microscopic pictures of histopathological variations in colorectal tissues for HE staining. *P＜0.05, **P＜0.01, ***P＜0.001

**Fig. 5:**
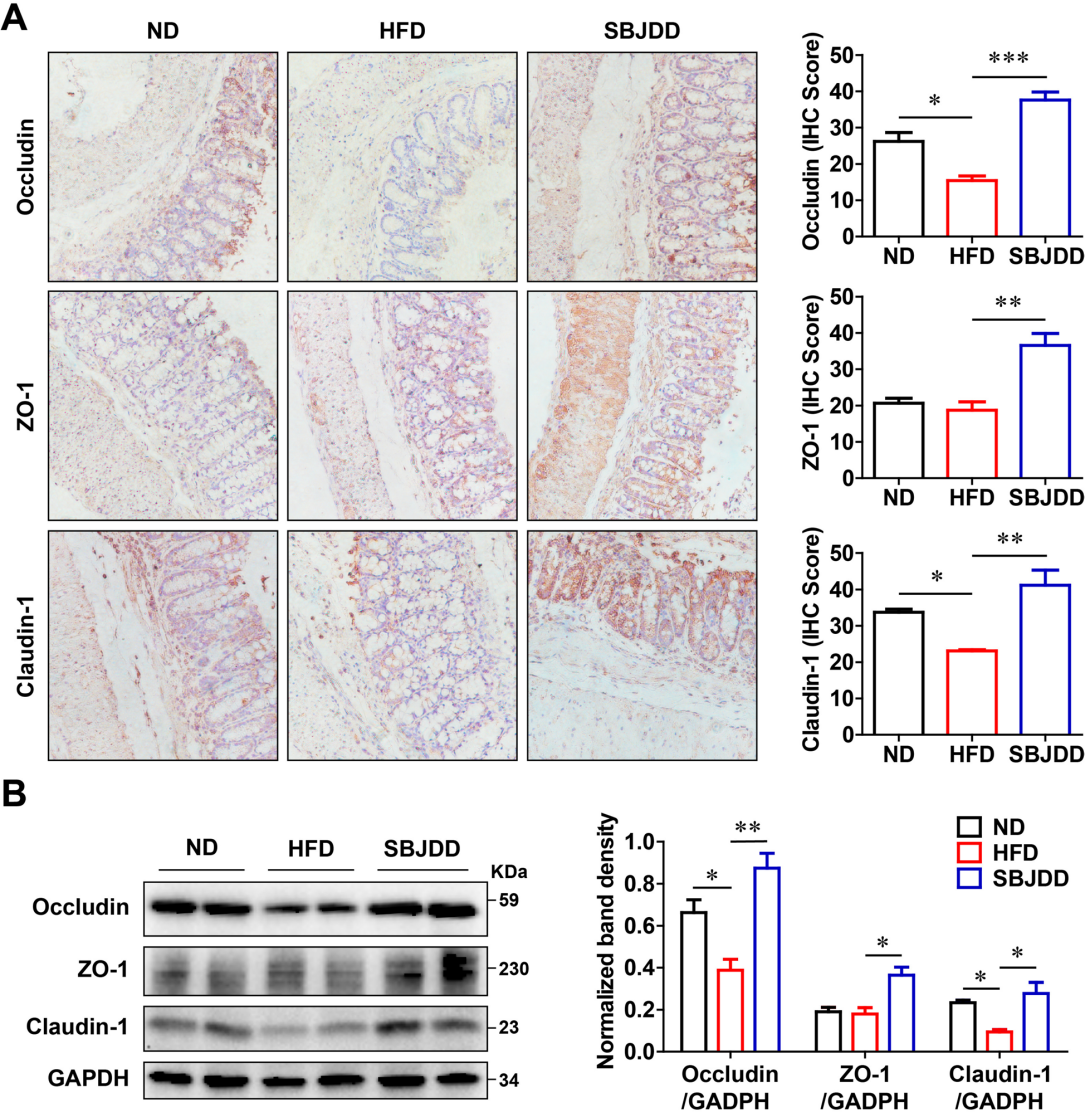
Effects of HFD and SBJDD administration on colorectal tight junction proteins associated with intestinal barrier function. **A** Representative microscopic pictures of colon tissues for IHC staining of Occludin, ZO-1, Claudin-1 (200 × magnification), and statistical histograms of the IHC score. **B** Western blot analysis of Occludin, ZO-1 and Claudin-1 in colorectal tissues. Data are shown as the mean ± SEM of at least three independent experiments. *P < 0.05, **P < 0.01, ***P < 0.001

To explore the efficacy of SBJDD in reducing colorectal adenoma formation and carcinogenesis, we utilized the Apc^min/+^ mice model and induced colorectal adenoma progression with a HFD. The schematic of the animal experiment was shown in Fig. 4A. The high-fat diet led to an increase in body weight prior to adenoma formation, whereas the subsequent loss of body weight during later stages of tumor development may be associated with the severity of cancer-related cachexia[21]. As seen from Fig. 4B, compared with the ND group, the HFD and SBJDD groups had higher body weight (*P* < 0.001). The HFD group started to lose weight rapidly at six weeks while the ND and SBJDD groups showed only a slight decrease in body weight. The HFD group resulted in a significant increase in the total tumor number (*P* < 0.05) and the number of tumors greater than 3 mm in diameter (*P* < 0.05). In line with this, HFD feeding significantly increased the incidence of colorectal carcinoma (*P* < 0.01). Whereas, SBJDD treatment effectively reversed this phenomenon (*P* < 0.01) (Fig. 4C–F). In addition, HE staining showed a more distorted cell arrangement, increased nucleus to cytoplasm ratio, and darker nuclear staining in the HFD group compared to the ND group. However, all the above phenomena were mitigated in the SBJDD group (Fig. 4G). Tumor progression can damage the intestinal barrier function and the expression of tight junction proteins is essential for intestinal barrier function. The down-regulation of tight junction proteins claudin-1, occludin, and ZO-1 may be associated with tumor proliferation, invasion, and metastasis [22, 23]. We evaluated the expression of claudin-1, occludin, and ZO-1 by immunohistochemistry and western blot. From Fig. 5A, B, we observed the down-regulated expression of claudin-1 and occludin in the HFD group compared to the ND group (*P* < 0.05) and SBJDD administration reversed the down-regulation effect of HFD (*P* < 0.01), an up-regulated expression of ZO-1 (*P* < 0.01) was also observed after SBJDD treatment. We also conducted validation experiments in the Apc^min/+^ mouse model without high-fat diet intervention to comprehensively evaluate the potential of SBJDD as a preventive approach for colorectal adenoma carcinogenesis. Our findings revealed that the SBJDD group exhibited a significant reduction in both tumor number and size (*P* < 0.05). Furthermore, SBJDD treatment significantly decreased the incidence of colorectal carcinoma (*P* < 0.01) (Fig. S1). The aforementioned findings collectively suggest that SBJDD may exert a suppressive effect on the formation of colorectal adenoma and carcinogenesis, while also safeguarding the integrity of the intestinal barrier.

### SBJDD regulated the gut microbiota and SCFAs, leading to the inhibition of tumor cell proliferation of Apc^min/+^ mice

**Fig. 6:**
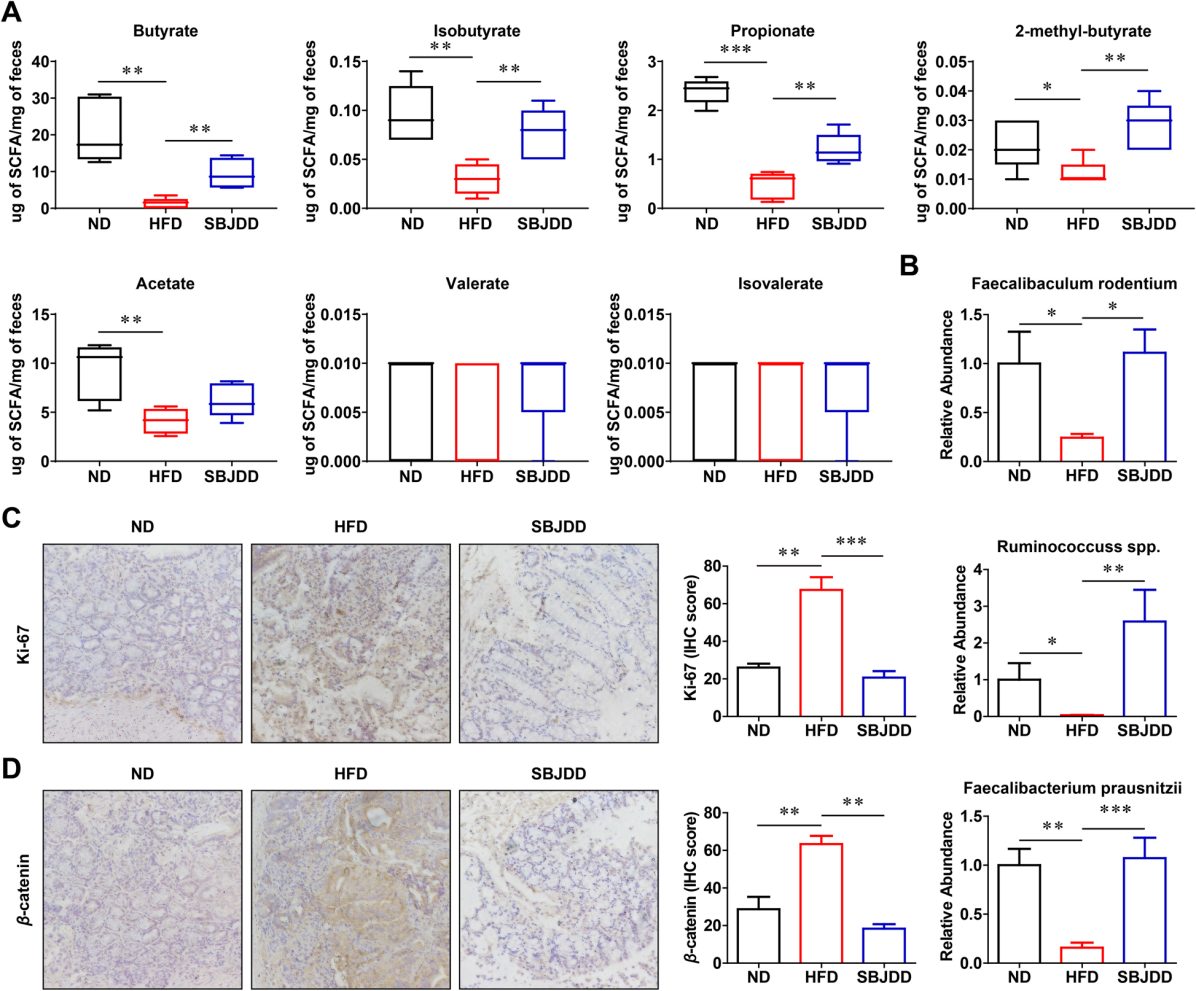
Effects of SBJDD administration on short-chain fatty acids in fecal samples and the expression of Ki67 and *β*-catenin in colorectal tissues. **A** The concentrations of acetate, butyrate, isobutyrate, propionate, 2-methyl-butyrate, valerate, and isovalerate in fecal samples of mice in ND (black, N = 5), HFD (red, N = 5), and SBJDD (blue, N = 5) groups. **B** The relative abundance of fecal representative gut microbiota in the three groups. **C**, **D** Representative microscopic pictures of colon tissues for IHC staining of Ki67 and β-catenin (200 × magnification) and statistical histograms of the positivity rate. Data are shown as the mean ± SEM of at least three independent experiments. *P < 0.05, **P < 0.01, ***P < 0.001

SCFAs are the major microbial fermentation products and are presumed to have a crucial role in host physiology. They have been demonstrated to mitigate intestinal inflammation, enhance the expression of tight junction proteins, and participate in the regulation of important signaling pathways [8]. Numerous studies have indicated that traditional Chinese medicine decoctions can elevate the levels of SCFAs in the intestine [24, 25]. Our clinical findings have shown that SBJDD can modulate the composition of gut microbiota and the production of SCFAs in patients with colorectal adenoma. Additionally, we collected mouse fecal samples for HPLC–MS/MS analysis to determine SCFAs content. As depicted in Fig. 6A, the HFD group exhibited a significant reduction in five out of seven SCFAs compared to the ND group (butyrate, isobutyrate, acetate: *P* < 0.01; 2-methyl-butyrate: *P* < 0.05; propionate: *P* < 0.001), SBJDD group had a higher content of four SCFAs than HFD group (butyrate, isobutyrate, 2-methyl-butyrate, propionate: *P* < 0.01), which indicated SBJDD can effectively reverse the HFD-induced decrease in SCFAs levels. In the previous 16S rRNA results, we observed a significant increase in the relative abundance of the genus Holdemanella in the post-treatment group. We also found the relative abundance of the species Faecalibaculum rodentium significantly increased in the SBJDD group (*P* < 0.05) compared to the HFD group. The species Faecalibaculum rodentium, a human homologue of Holdemanella biformis within the genus Holdemanella, has been shown to have a reduction in advanced adenoma stages. Additionally, it is able to inhibit tumor cell growth in vitro through the release of SCFAs [26]. The relative abundance of Ruminococcuss spp. (*P* < 0.01) and Faecalibacterium prausnitzii (*P* < 0.001), both belonging to Firmicutes [19], known for their production of SCFAs, also exhibited an increase in the SBJDD group. The qPCR results are consistent with our previous findings, providing further evidence of the role of SBJDD in modulating gut microbiota that is capable of producing SCFAs (Fig. 6B).

Next, we used immunohistochemistry assay to assess the expression of Ki-67 and β-catenin in colorectal tissues. The results showed that HFD significantly increased the proliferation marked by Ki67 (*P* < 0.01) and SBJDD administration restored proliferation levels to a similar level to the ND group (*P* < 0.001). Activation of β-catenin is crucial in the progression of colorectal tumors and may contribute to increased cell proliferation, posing a risk factor for colorectal adenoma-carcinoma sequence [27], HFD drove elevated expression of *β*-catenin, while SBJDD significantly reduced the expression of *β*-catenin (Fig. 6C, D, P < 0.01).

### SBJDD activated G protein-coupled receptors, inhibited histone deacetylases

**Fig. 7:**
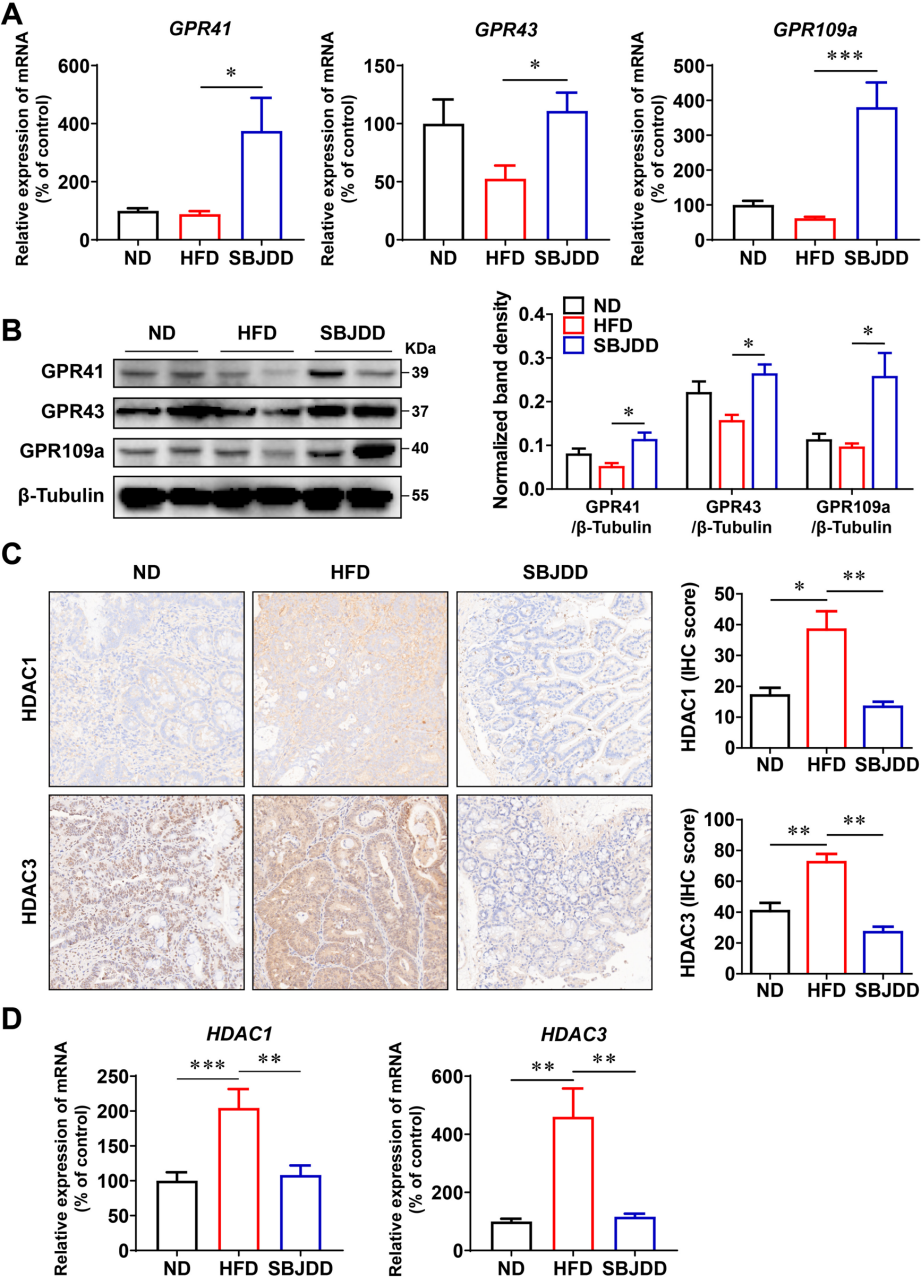
Comparison of expressions of HDAC1/3 and GPR41/43/109a in colorectal tissue of mice among ND, HFD and SBJDD groups. **A** GPR41, GPR43 and GPR109a relative expression of mRNA in colon tissues were measured by RT-qPCR analysis. **B** Western blot analysis of GPR41, GRP43 and GPR109a in colorectal tissues. **C** Representative microscopic pictures of colorectal tissues for IHC staining of HDAC1 and HDAC3. **D** HDAC1 and HDAC3 relative expression of mRNA in colon tissues were measured by RT-qPCR. Data are shown as the mean ± SEM of at least three independent experiments. *P < 0.05, **P < 0.01, ***P < 0.001

Multiple studies have demonstrated the direct agonistic effects of SCFAs on G protein-coupled receptors (GPR) and their inhibitory effects on histone deacetylases (HDAC), leading to the inhibition of colorectal cancer growth [8, 28]. Given our findings regarding the promotion of SCFA production by SBJDD, we hypothesized that SBJDD may activate GPRs and inhibit HDACs. To verify this hypothesis, we conducted RT-PCR analysis, immunohistochemistry, and western blot assays. From the results of RT-qPCR (Fig. 7A), there was no significant difference in the expression of GPR41, GPR43 and GPR109a between the HFD and ND groups, while SBJDD administration significantly increased the relative expression levels of GPR41, GPR43 (*P* < 0.05) and GPR109a (*P* < 0.001). The western blot results also demonstrated that relative protein expression levels of GPR41, GPR43 and GPR109a significantly elevated in the SBJDD group compared to the HFD group (Fig. 7B, P < 0.05). These results suggested that SBJDD has a certain activation effect on GPRs. In the HFD group, intense HDAC1 and HDAC3 immunostaining was detected, and diminished immunostaining was observed in the SBJDD group compared to the HFD group (Fig. 7C, P < 0.01). The relative mRNA expression of HDAC1 and HDAC3 was in full agreement with the immunohistochemistry results, with HFD strongly increasing HDAC1 and HDAC3 expression while SBJDD administration reversing this trend (Fig. 7D, P < 0.01).

### SBJDD regulated the expression of inflammatory factors in colorectal tissue and induced M2-like macrophage polarization

**Fig. 8:**
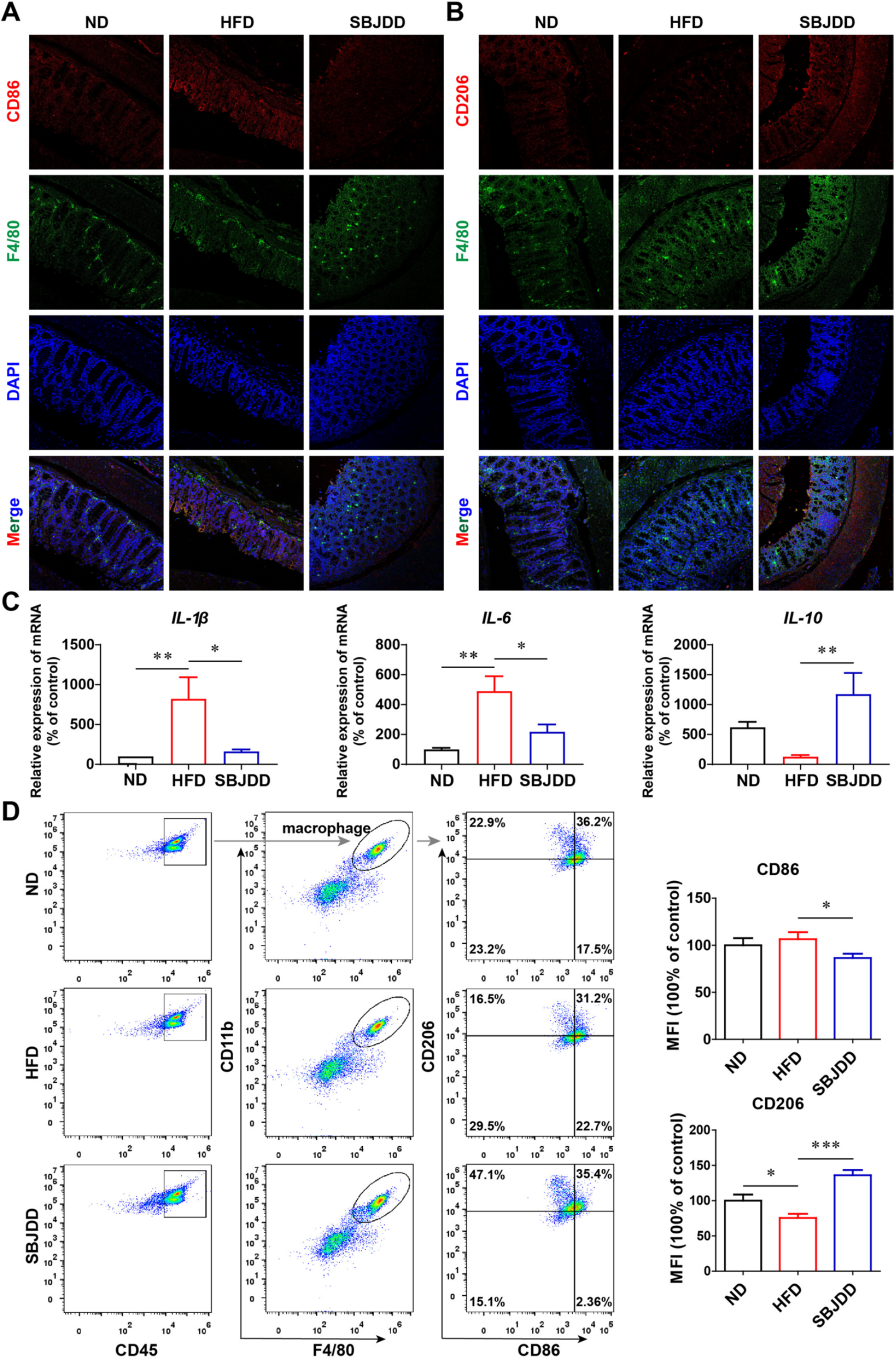
Effects of SBJDD on secretion of inflammatory factors and polarization of tumor-associated macrophages in the mouse colorectum. **A**, **B** Multicolor immunofluorescence analysis of tumor-associated macrophages on mouse colorectal tissues of each group (F4/80: green, CD206: red, CD86: red, DAPI: blue; 200 × magnification). **C** The relative expression of IL-1β, IL-6, and IL-10 mRNA in colorectal tissues of each group was measured by RT-qPCR. **D** The proportion of M1-type macrophages and M2-type macrophages in peritoneal lavage fluid of each group, and the average fluorescence intensity of CD86 and CD206 in each group. Data are shown as the mean ± SEM of at least three independent experiments. *P < 0.05, **P < 0.01, ***P < 0.001

HFD promotes intestinal inflammation and induces macrophages towards M1 polarization, while SCFAs have been reported to exert anti-inflammatory effects by activating G-protein-coupled receptor signaling and inhibiting histone deacetylases [29]. Notably, macrophages have two polarized phenotypes, the pro-inflammatory M1 type and the anti-inflammatory M2 type. Macrophages of the M1 phenotype can promote the secretion of inflammatory factors such as IL-1β and IL-6 [30]. SCFAs have an impact on the polarization of macrophages [31]. We first used immunofluorescent staining of F4/80 (macrophages marker), CD86 (M1-type macrophages marker), and CD206 (M2-type macrophages marker) to detect the polarization of macrophages on mouse colorectal tissues, as shown in Fig. 8A, B, the fluorescence intensity of CD86 was significantly enhanced in the tissues of the HFD group compared to the ND group, while the fluorescence intensity of CD206 was significantly weakened, indicating that HFD promoted macrophage polarization towards M1 type. Although the mice in the SBJDD group were also HFD-fed, the fluorescence intensity of CD86 was significantly weaker than that of the HFD group and the fluorescence intensity of CD206 was enhanced, suggesting that the polarization of macrophages may have been reversed by the administration of SBJDD. The relative expression of mRNA in colorectal tissues showed that expression of pro-inflammatory cytokines IL-1β and IL-6 were significantly higher in the HFD group (*P* < 0.05, *P* < 0.001), the relative expression of these two pro-inflammatory factors was significantly down-regulated in the SBJDD group, and the relative expression of the anti-inflammatory cytokine IL-10 was significantly up-regulated (Fig. 8C, P < 0.05, *P* < 0.05, *P* < 0.01). In the SBJDD group, the proportion of M1-type macrophages significantly decreased and M2-type macrophages increased in peritoneal lavage fluid. The mean fluorescence intensity of CD86 significantly decreased and CD206 increased (Fig. 8D, P < 0.05, *P* < 0.001). Therefore, SBJDD can exert an anti-inflammatory effect by regulating macrophage polarization.

## Discussion

SBJDD is an empirical prescription developed by Professor Cheng Haibo and has been clinically used to treat colorectal adenomas for years. We conducted a multicenter, randomized, double-blind, placebo-controlled clinical trial to evaluate the efficacy of SBJDD at eight hospitals in China. Our results demonstrated that SBJDD significantly reduced the recurrence rate of colorectal adenoma by 16.07% while maintaining a high level of safety [6]. However, the mechanism of SBJDD to inhibit adenoma carcinogenesis is still not well understood and remained to be elucidated. In this research, we adopted the Apc^min/+^ mice model and accelerated colorectal adenoma progression by the HFD to assess the pharmacological effects of SBJDD. The results of this study indicated that SBJDD may exert its inhibitory effects on colorectal adenoma carcinogenesis by regulating gut microbiota composition, promoting the production of SCFAs, inducing M2-like macrophage polarization, reducing intestinal inflammation, inhibiting cancer cell proliferation and restoring intestinal barrier function.

Our clinical study has demonstrated that SBJDD can modulate the composition of gut microbiota and enhance the production of SCFAs in patients with colorectal adenoma. We focused on the changes in the family of firmicute that can produce SCFAs [20] before and after the administration of SBJDD. 16S rRNA gene sequence analysis of patients' fecal samples revealed a significant increase in several genera in the family of Firmicute after administration of SBJDD, including Christensenellaceae R-7 group, Holdemanella, and Anaerotruncus. These results may imply that SBJDD can influence metabolites by regulating gut microbiota. In order to investigate the efficacy of SBJDD in reducing colorectal adenoma formation and carcinogenesis, we employed the Apc^min/+^ mice model and induced colorectal adenoma progression with a HFD. HFD is frequently associated with obesity and represents a potential risk factor for colorectal tumors through various pathways, including disruption of the original balance of intestinal microbes, disturbance of intestinal metabolism, and induction of inflammation [32, 33]. HFD also can directly act on intestinal progenitors and stem cells, leading to an elevated possibility of intestinal tumor development [34]. Mice in the HFD group exhibited a higher incidence of colorectal tumors, an increased prevalence of colorectal carcinoma, and more severe inflammation, while SBJDD ameliorated these phenomena. We also conducted validation experiments in the Apc^min/+^ mouse model without high-fat diet intervention to comprehensively evaluate the potential of SBJDD as a preventive approach for colorectal adenoma carcinogenesis. Our findings revealed that the SBJDD group exhibited a significant reduction in both tumor number and size. Furthermore, SBJDD treatment significantly decreased the incidence of colorectal carcinoma. The aforementioned findings collectively suggest that SBJDD may exert a suppressive effect on the formation of colorectal adenoma and carcinogenesis, while also safeguarding the integrity of the intestinal barrier.

A healthy diet with a higher intake of dietary fiber should be advocated, intestinal bacteria can ferment dietary fiber to produce SCFAs, which plays a crucial role in the prevention of colorectal cancer [8]. Our study has demonstrated that SBJDD can modulate gut microbiota that is capable of producing SCFAs. The content of SCFAs, such as acetate, butyrate, isobutyrate, and propionate, were significantly upregulated in the SBJDD group. These results were also consistent with the clinical results, with increased levels of SCFAs in the patients' samples following administration of SBJDD, suggesting that SBJDD may exert its inhibitory effects on colorectal adenoma carcinogenesis by regulating gut microbiota and SCFAs.

Interestingly, we found considerably lower levels of SCFAs in patient serum samples than in fecal samples, and we speculate that the inhibitory effect of SCFAs on tumor development is mainly through direct action on the intestinal epithelial, but this remains to be further confirmed. The current study suggests that SCFAs produced by bacterial fermentation are absorbed by absorptive epithelial cells and can regulate G protein-coupled receptors expressed in colonic epithelial cells including GPR41, GPR43 and GPR109a [35–37]. Additional study has reported that butyrate can also promote epithelial barrier function by acting on the IL-10 receptor [38]. Amazingly, our results unveiled that SBJDD activated GPR41, GPR43, and GPR109a and increased the expression of IL-10. SCFAs can also serve as HDAC inhibitors [39]. T-cell differentiation can be directly facilitated by SCFAs through the inhibition of histone deacetylase [40]. In a study of the effects of butyrate on human intestinal epithelial cells, its influence was imitated by Trichostatin A, a specific inhibitor of HDAC class I/II [41]. We found decreased expression of HDAC in the colorectal tissues after administration of SBJDD. These results were consistent with those reported in previous studies and suggest that SBJDD may be effective by increasing the production of SCFAs and activating G protein-coupled receptors, inhibiting histone deacetylases.

Previous studies have highlighted the crucial role of gut microbiota in the development of intestinal inflammation and malignant intestinal diseases [42]. It is well-established that chronic inflammation serves as a risk factor for colorectal cancer [43],

while SCFAs have been shown to possess anti-inflammatory properties [44, 45]. In this study, SBJDD was found to down-regulate the relative expression of pro-inflammatory cytokines and up-regulate the anti-inflammatory cytokine. Additionally, SBJDD demonstrated a capacity to decrease the proportion of M1-type macrophages while increasing the proportion of M2-type macrophages. Overall, these findings suggest that SBJDD has potential in ameliorating inflammatory tendencies in the colorectum of mice, which may play a significant role in inhibiting colorectal adenoma progression.

Our study objectively demonstrated the pharmacological effects of SBJDD in inhibiting the progression of colorectal adenoma and investigated its mechanisms in terms of regulating gut microbiota, increasing SCFAs, and reducing colorectal inflammation. These findings provide a basis for the clinical application of SBJDD and lay a foundation for further research in this area. The current study is undergoing further refinement, with ongoing efforts to expand the cohort of colorectal adenoma patients and to explore more specific components and targets for SBJDD in order to enhance its pharmacological efficacy in future investigations.

## Conclusions

**Fig. 9:**
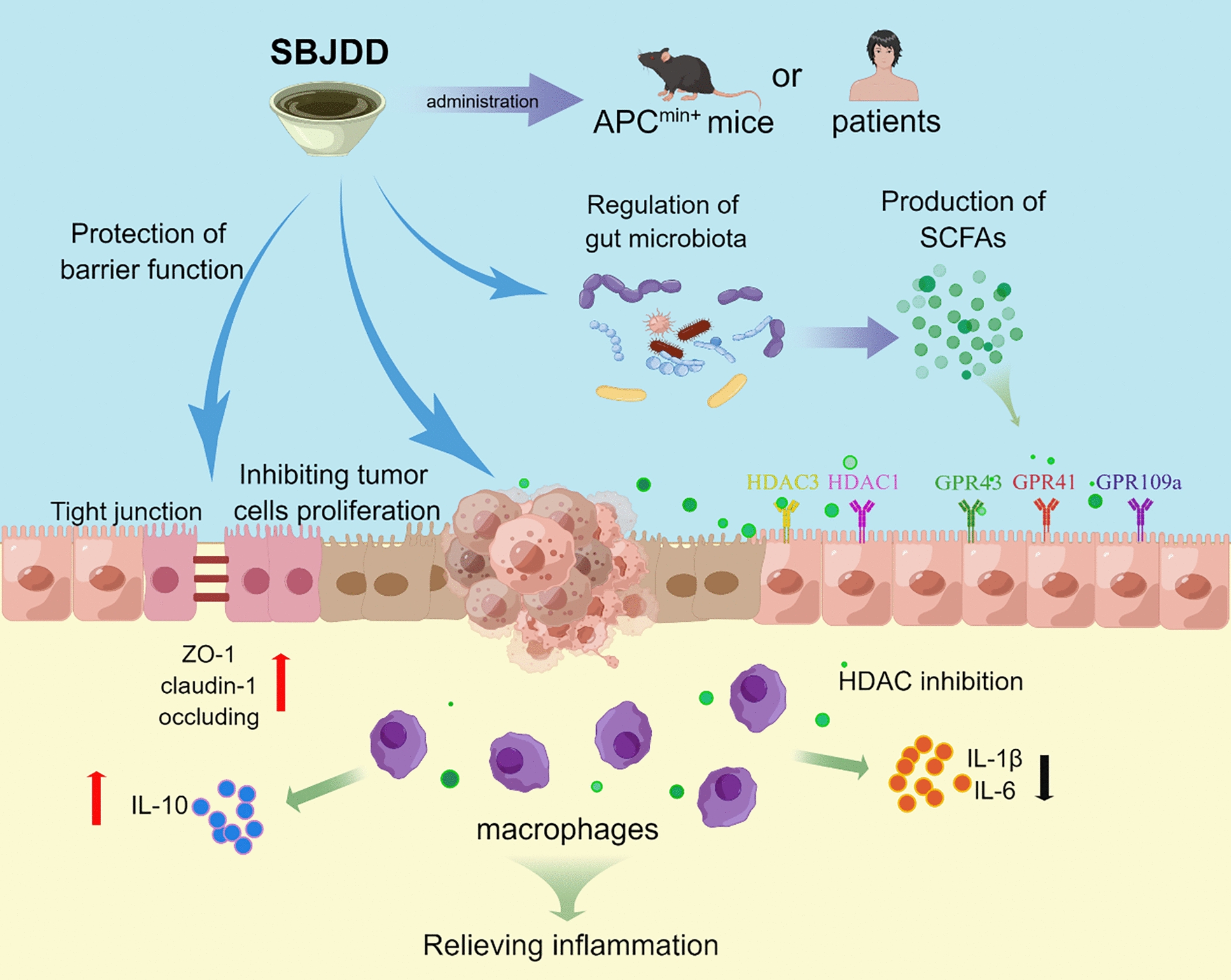
The schematic diagram of the mechanism of SBJDD. SBJDD may exert its inhibitory effects on colorectal adenoma carcinogenesis by regulating gut microbiota, promoting the production of SCFAs, activating G protein-coupled receptors GPR43, GPR41 and GPR109a, inhibiting histone deacetylases HDAC1 and HDAC3, inducing M2-like macrophage polarization, reducing intestinal inflammation, inhibiting cancer cell proliferation and restoring intestinal barrier function

Collectively, our study indicated that SBJDD may exert its inhibitory effects on colorectal adenoma carcinogenesis by regulating gut microbiota, promoting the production of SCFAs, activating G protein-coupled receptors GPR43, GPR41 and GPR109a, inhibiting histone deacetylases HDAC1 and HDAC3, inducing M2-like macrophage polarization, reducing intestinal inflammation, inhibiting cancer cell proliferation and restoring intestinal barrier function. A schematic diagram of the mechanism of SBJDD in inhibiting the progression of colorectal adenoma to carcinoma progression is shown in Fig. 9.

## Experiments limitations

Our clinical study has demonstrated that SBJDD significantly reduced the recurrence of adenomas. The single dose used in our experiment was converted according to the clinical dosage. Currently, there is no widely recognized positive control for the clinical treatment of colorectal adenoma. However, it should be noted that the single dose utilized in this study and the absence of positive controls do present certain limitations, which will be addressed and improved upon in future studies.

## Supplementary Information

Additional file 1. Figure S1. Effects of SBJDD administration on spontaneously form colorectal adenomas model of Apc^min/+^ mice. **A** Schematic of in vivo experimental procedure. **B** Body weight change. **C** Representative colon appearances. **D** Statistical graph of a total number of tumors. Ctrland C-SBJDD. **E** Statistical graph of a total number of tumors diameter larger than 3 mm and smaller than 3 mm. **F** Representative microscopic pictures of histopathological variations in colorectal tissues for HE staining. **G** The quantitative analysis of the pathologic score was determined based on the following criteria: 0 for normal, 1 for low-grade dysplasia, 2 for high-grade dysplasia, and 3 for carcinoma

Additional file 2

Additional file 3

Additional file 4

## Data Availability

The datasets analyzed during this study are available from the corresponding author on reasonable request.

## Abbreviations

SCFAs: Short-chain fatty acids
HDAC: Histone deacetylases
GPCR: G-protein coupled receptors
ND: Normal diet
HFD: High-fat diet
SBJDD: Shen-Bai-Jie-Du decoction
IF: Immunofluorescent staining
CRC: Colorectal cancer
RT-qPCR: Real-time quantitative PCR analysis
HE: Hematoxylin and eosin staining
IHC: Immunohistochemistry
IL-1*β*: Interleukin 1 beta
IL-6: Interleukin 6
IL-10: Interleukin 10
APC: Adenomatous polyposis coli
