# Supplementary material for: Shen-Bai-Jie-Du decoction suppresses the progression of colorectal adenoma to carcinoma through regulating gut microbiota and short-chain fatty acids
Source: Chin Med. 2024 Oct 28;19:149. doi: 10.1186/s13020-024-01019-4 (PMC11514841; doi:10.1186/s13020-024-01019-4)
Supplement: Supplementary file 1 — Additional file 1. Figure S1. Effects of SBJDD administration on spontaneously form colorectal adenomas model of Apcmin/+ mice. A Schematic of in vivo experimental procedure. B Body weight change. C Representative colon appearances. D Statistical graph of a total number of tumors. Ctrland C-SBJDD. E Statistical graph of a total number of tumors diameter larger than 3 mm and smaller than 3 mm. F Representative microscopic pictures of histopathological variations in colorectal tissues for HE staining. G The quantitative analysis of the pathologic score was determined based on the following criteria: 0 for normal, 1 for low-grade dysplasia, 2 for high-grade dysplasia, and 3 for carcinoma [file 13020_2024_1019_MOESM1_ESM.docx]

**Mice and establishment of model**

The experiments were conducted under the supervision and guidance of the Experimental Animal Ethics Committee of Nanjing University of Chinese Medicine, in accordance with the requirements of experimental animal ethics (Ethic code: 202310A062). At 4 weeks of age, Apc^min/+^ mice (strain NO. T001457) were procured from Jiangsu GemPharmatech (Jiangsu, China) and housed in the Experimental Animal Centre of Nanjing University of Chinese Medicine. The housing conditions included a relative humidity of 55±15%, an ambient temperature of 23±3℃, and a 12-hour light/dark cycle, with ad libitum access to food and water.

After one week of the adaptation period, sixteen mice were randomly allocated into two groups (N=8). The two groups are Ctrl group (continuous maintenance feed and intragastric administration of saline), and C-SBJDD group (continuous maintenance feed and intragastric administration of SBJDD). The intragastric administration of SBJDD in mice was performed at a dose of approximately 14.3 g/kg per day, equivalent to the human equivalent dose. The SBJDD and saline were administered six consecutive days in one week. The body weight of mice was measured every two days. After 12 weeks, the mice were sacrificed, the colorectal tissue of the mice was dissected along the longitudinal axis, the number, size, and location of adenomas were recorded. Part of the colorectal tissue was fixed in 4% paraformaldehyde and then made into paraffin-embedded tissue sections for HE staining.


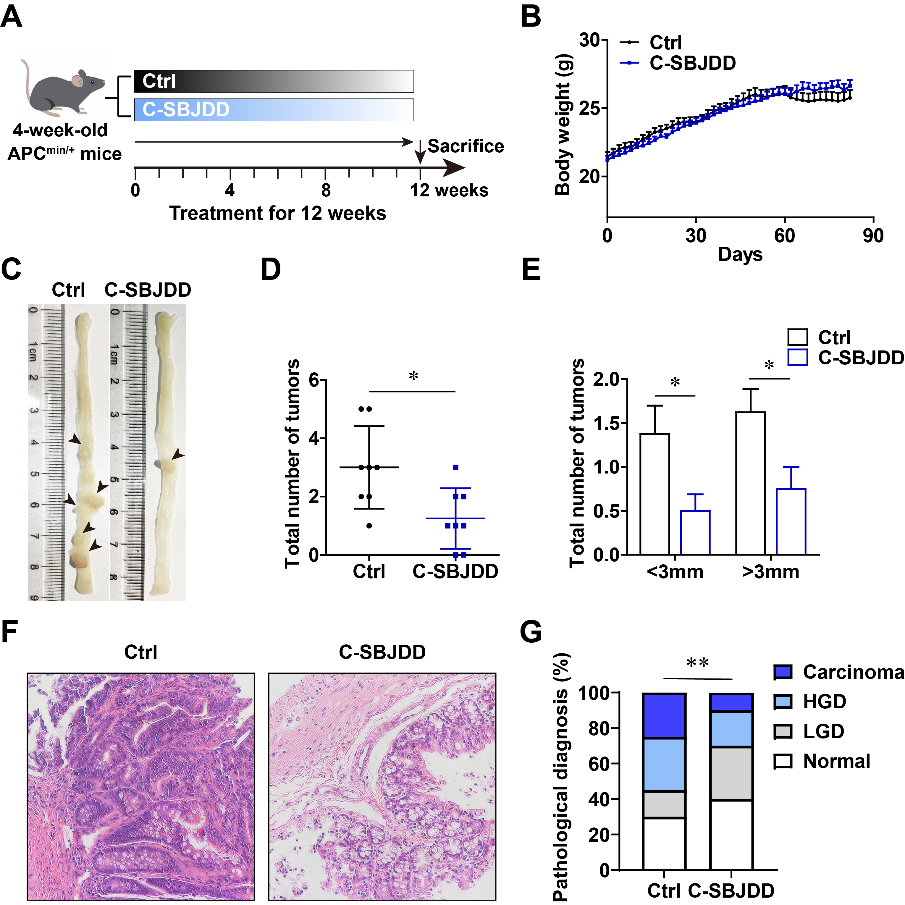


**Fig. S1 Effects of SBJDD administration on spontaneously form colorectal adenomas model of Apc^min/+^ mice.** (**A**) Schematic of in vivo experimental procedure (Ctrl: normal diet group, C-SBJDD: Shen-Bai-Jie-Du decoction group with normal diet). (**B**) Body weight change. (**C**) Representative colon appearances. (**D**) Statistical graph of a total number of tumors. Ctrl (black, N=8) and C-SBJDD (blue, N=8). (**E**) Statistical graph of a total number of tumors diameter larger than 3 mm and smaller than 3 mm. (**F**) Representative microscopic pictures of histopathological variations in colorectal tissues for HE staining (200×magnification). (**G**) The quantitative analysis of the pathologic score was determined based on the following criteria: 0 for normal, 1 for low-grade dysplasia (LGD), 2 for high-grade dysplasia (HGD), and 3 for carcinoma.


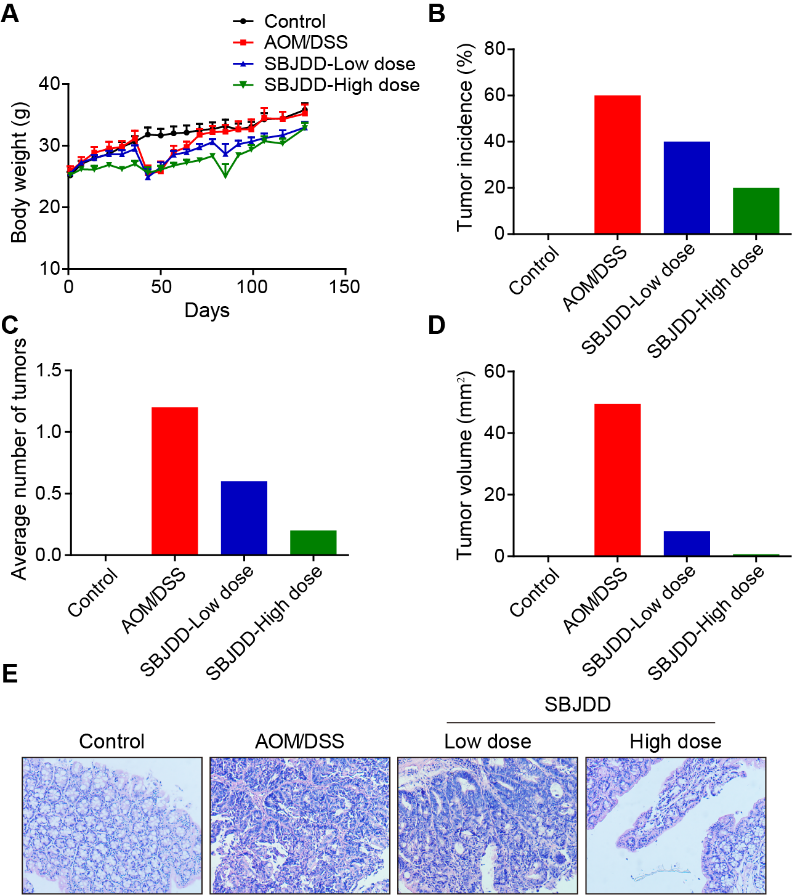


**Fig. S2 SBJDD inhibits AOM/DSS-induced colorectal adenoma formation and carcinogenesis in C57BL/6J mice.** (**A**) Body weight change. (**B-D**) Tumor incidence (**B**) number of tumors formed (**C**) and tumor volume (**D**) in each group. (**E**) Formation of adenoma and adenocarcinoma in each group (HE staining, original magnification: 200).
