# Supplementary material for: Shen-Bai-Jie-Du decoction suppresses the progression of colorectal adenoma to carcinoma through regulating gut microbiota and short-chain fatty acids
Source: Chin Med. 2024 Oct 28;19:149. doi: 10.1186/s13020-024-01019-4 (PMC11514841; doi:10.1186/s13020-024-01019-4)
Supplement: Supplementary file 2 — Additional file 2 [file 13020_2024_1019_MOESM2_ESM.docx]

**Supplementary Table 1. The basic information and pathological conditions of patients enrolled in the research.**

| **Number** | **Age** | **Gender** | **Pathological conditions** | | |
| --- | --- | --- | --- | --- | --- |
|  |  |  | **Non-tubular/Tubular/ Villous-tubular** | **Low-grade/High-grade intraepithelial neoplasia** | **Mild /Moderate to severe/Severe dysplasia** |
| 1 | 63 | male | Tubular | Low-grade | Mild |
| 2 | 56 | male | Villous-tubular | Low-grade | Mild |
| 3 | 57 | male | Tubular | Low-grade | Mild |
| 4 | 55 | male | Tubular | High-grade | Moderate to severe |
| 5 | 39 | male | Tubular | Low-grade | Mild |
| 6 | 52 | female | Tubular | Low-grade | Mild |
| 7 | 32 | male | Non-tubular | Low-grade | Mild |
| 8 | 68 | female | Tubular | Low-grade | Mild |
| 9 | 66 | male | Tubular | Low-grade | Mild |
| 10 | 56 | male | Tubular | Low-grade | Mild |
| 11 | 55 | male | Tubular | High-grade | Severe |
| 12 | 67 | male | Tubular | High-grade | Moderate to severe |
| 13 | 42 | female | Tubular | High-grade | Mild |
| 14 | 50 | male | Tubular | High-grade | Moderate to severe |
| 15 | 56 | female | Villous-tubular | Low-grade | Mild |
| 16 | 70 | male | Villous-tubular | High-grade | Moderate to severe |
| 17 | 57 | male | Villous-tubular | Low-grade | Mild |
| 18 | 58 | male | Villous-tubular | Low-grade | Mild |
| 19 | 63 | male | Tubular | Low-grade | Mild |
| 20 | 59 | male | Villous-tubular | Low-grade | Mild |
| 21 | 56 | female | Villous-tubular | Low-grade | Mild |
