# Supplementary material for: Shen-Bai-Jie-Du decoction suppresses the progression of colorectal adenoma to carcinoma through regulating gut microbiota and short-chain fatty acids
Source: Chin Med. 2024 Oct 28;19:149. doi: 10.1186/s13020-024-01019-4 (PMC11514841; doi:10.1186/s13020-024-01019-4)
Supplement: Supplementary file 3 — Additional file 3 [file 13020_2024_1019_MOESM3_ESM.docx]

**Supplementary Table 2. Detailed information of the content components of SBJDD detected by UPLC-MS/MS analysis.**

| **No** | **Name** | **Formula** | **m/z** | **Retention time (min)** | **Ratio of mean peak area (%) (N=3)** |
| --- | --- | --- | --- | --- | --- |
| 1 | Kushenol O | C27H30O13 | 563.1753093 | 5.301633333 | 13.41628388 |
| 2 | Trifolirhizin | C22H22O10 | 491.1193506 | 5.980216667 | 9.988172693 |
| 3 | Melezitose | C18H32O16 | 527.1579555 | 0.965216667 | 7.305143089 |
| 4 | Cryptochlorogenic acid | C16H18O9 | 353.0877726 | 4.18015 | 5.085401901 |
| 5 | Methyl deacetylasperulosidate | C17H24O11 | 449.1300546 | 3.651816667 | 4.968583278 |
| 6 | Glucose | C6H12O6 | 225.0614693 | 0.834716667 | 4.573550738 |
| 7 | Neochlorogenic acid | C16H18O9 | 353.0878325 | 3.760383333 | 3.8314661 |
| 8 | Phlorigidoside C | C17H24O11 | 427.1212903 | 3.70465 | 3.319879646 |
| 9 | Methyl citrate | C7H10O7 | 205.0354051 | 1.984266667 | 2.807599699 |
| 10 | Vitexin-2"-O-rhamnoside | C27H30O14 | 579.1715207 | 4.71775 | 2.739841384 |
| 11 | N-Acetylmannosamine | C8H15NO6 | 204.0867 | 0.94998333 | 2.170061868 |
| 12 | 3-Hydroxyphenylalanine | C9H11NO3 | 182.0813 | 1.30358333 | 2.090894644 |
| 13 | Lobetyolin | C20H28O8 | 441.1765 | 5.39321667 | 1.97070456 |
| 14 | Sesamoside | C17H24O12 | 419.1197 | 3.96068333 | 1.756162118 |
| 15 | Quercetin-3-O-D-glucosyl]-(1-2)-L-rhamnoside | C27H30O16 | 609.1463 | 4.77258333 | 1.715211803 |
| 16 | Asperulosidic Acid | C18H24O12 | 431.1195 | 3.99708333 | 1.439960572 |
| 17 | Turanose | C12H22O11 | 360.1497 | 0.93471667 | 1.426301479 |
| 18 | 1,4-D-Gulonolactone | C6H10O6 | 223.0458 | 0.9288 | 1.254247226 |
| 19 | 3-Feruloylquinic acid | C17H20O9 | 735.2142 | 4.62956667 | 1.116451769 |
| 20 | Apiin | C26H28O14 | 565.1558 | 4.87366667 | 1.056236352 |
| 21 | Chlorogenic acid | C16H18O9 | 355.1029 | 4.21883333 | 0.973818263 |
| 22 | Glycitein | C16H12O5 | 283.0611 | 6.20148333 | 0.917612023 |
| 23 | Manosamine | C6H13NO5 | 162.0761 | 0.88901667 | 0.917131584 |
| 24 | Shogaol | C17H24O3 | 277.1798 | 10.5671333 | 0.897472947 |
| 25 | Puerarin 6''-O-Xyloside | C26H28O13 | 593.1511 | 4.21663333 | 0.856902905 |
| 26 | Atractylenolide III | C15H20O3 | 231.138 | 9.53301667 | 0.800421836 |
| 27 | Baimaside | C27H30O17 | 627.1558 | 4.50718333 | 0.796535525 |
| 28 | Oroxylin A-7-O-glucoside | C22H22O10 | 447.1281 | 6.00243333 | 0.779819661 |
| 29 | Prunasin | C14H17NO6 | 340.1036 | 4.64648333 | 0.776850221 |
| 30 | Negletein | C16H12O5 | 285.0757 | 8.3357 | 0.650014917 |
| 31 | Cassiaside B | C26H30O14 | 565.1562 | 5.52151667 | 0.610606218 |
| 32 | Dalbergin | C16H12O4 | 267.0661 | 7.81995 | 0.610362469 |
| 33 | Asperuloside | C18H22O11 | 459.1145 | 4.306 | 0.54535348 |
| 34 | Atractyloside A | C21H36O10 | 493.2291 | 3.92381667 | 0.542879712 |
| 35 | Pinoresinol 4-O-beta-D-glucopyranoside | C26H32O11 | 565.1928 | 5.0629 | 0.540838795 |
| 36 | Alizarin 1-methyl ether | C15H10O4 | 255.0652 | 7.92856667 | 0.538425108 |
| 37 | Oxyberberine | C20H17NO5 | 374.0998 | 9.23603333 | 0.513514911 |
| 38 | N-Acetylarginine | C8H16N4O3 | 217.1296 | 1.0724 | 0.477129108 |
| 39 | Calycosin 7-galactoside | C22H22O10 | 447.129 | 4.85638333 | 0.458637363 |
| 40 | Anthraquinone-2-carboxylic acid | C15H8O4 | 251.0349 | 8.03725 | 0.458565063 |
| 41 | Lobetyolinin | C26H38O13 | 603.2298 | 5.00821667 | 0.452222397 |
| 42 | Ononin | C22H22O9 | 475.1243 | 5.59541667 | 0.42189388 |
| 43 | Neoamygdalin | C20H27NO11 | 502.1566 | 4.27081667 | 0.383201459 |
| 44 | Atractylenolide II | C15H20O2 | 233.1536 | 10.5671333 | 0.366073394 |
| 45 | Scopoletin | C10H8O4 | 193.0497 | 5.20108333 | 0.364040956 |
| 46 | Allitol | C6H14O6 | 181.0718 | 0.83471667 | 0.358708738 |
| 47 | 3-Demethylcolchicine | C21H23NO6 | 368.1495 | 5.11808333 | 0.357308248 |
| 48 | Hexahydrocurcumin | C21H26O6 | 357.1696 | 6.73773333 | 0.319789504 |
| 49 | Fusaric acid | C10H13NO2 | 180.1021 | 4.92676667 | 0.316120382 |
| 50 | 2-Hydroxy-3-(hydroxymethyl)anthraquinone | C15H10O4 | 253.0506 | 6.62593333 | 0.300877531 |
| 51 | Quercetin 3-sambubioside | C26H28O16 | 595.1305 | 4.610833333 | 0.299690145 |
| 52 | Swertisin | C22H22O10 | 491.1196 | 4.84395 | 0.294388635 |
| 53 | Rutin | C27H30O16 | 611.1617 | 4.752133333 | 0.275874562 |
| 54 | ClemastaninB | C32H44O16 | 729.261 | 4.216633333 | 0.259836218 |
| 55 | Roseoside | C19H30O8 | 431.1923 | 4.50245 | 0.25843876 |
| 56 | Vanillic acid 4-beta-D-glucoside | C14H18O9 | 329.0876 | 3.212066667 | 0.251040647 |
| 57 | 7-[(beta-D-Glucopyranosyl)oxy]-3',4',5,8-tetrahydroxyflavone | C21H20O12 | 463.0884 | 4.8621 | 0.219391466 |
| 58 | Sibiricose A6 | C23H32O15 | 529.1562 | 4.18015 | 0.197950773 |
| 59 | 5-Acetylsalicylic acid | C9H8O4 | 179.0352 | 5.026966667 | 0.196513126 |
| 60 | Genistein | C15H10O5 | 269.0455 | 6.828183333 | 0.1910104 |
| 61 | Salutaridine | C19H21NO4 | 328.1547 | 4.507183333 | 0.171996723 |
| 62 | Demethyltexasin | C15H10O5 | 269.0454 | 5.374683333 | 0.17188699 |
| 63 | Levoglucosan | C6H10O5 | 163.06 | 0.965216667 | 0.17148146 |
| 64 | Syringaresinol | C22H26O8 | 401.1596 | 6.357416667 | 0.16227154 |
| 65 | Tabersonine | C21H24N2O2 | 319.1806 | 6.002433333 | 0.155337592 |
| 66 | Isoxanthohumol | C21H22O5 | 353.1393 | 7.96505 | 0.154405522 |
| 67 | Bombykol | C16H30O | 256.2634 | 12.62553333 | 0.149672358 |
| 68 | Aurantiamide acetic acid | C27H28N2O4 | 467.194 | 9.8657 | 0.136202777 |
| 69 | Isoquercitrin | C21H20O12 | 465.1034 | 4.891383333 | 0.131586823 |
| 70 | 5-Feruloylquinic acid | C17H20O9 | 735.2141 | 4.270816667 | 0.125060852 |
| 71 | Flazin | C17H12N2O4 | 309.0871 | 6.394066667 | 0.124807523 |
| 72 | Morin | C15H10O7 | 301.0353 | 6.1092 | 0.123139825 |
| 73 | 19-Oxocinobufotalin | C26H32O8 | 473.2171 | 6.883116667 | 0.122999978 |
| 74 | 4-O-beta-Glucopyranosyl-cis-coumaric acid | C15H18O8 | 371.0985 | 3.8508 | 0.109105603 |
| 75 | 3-epi-Padmatin | C16H14O7 | 299.056 | 6.348883333 | 0.107708701 |
| 76 | Octahydrocurcumin | C21H28O6 | 377.1951 | 6.321433333 | 0.096681436 |
| 77 | Syringaldehyde | C9H10O4 | 183.0654 | 5.1507 | 0.096054142 |
| 78 | Episyringaresinol 4'-O-beta-D-glncopyranoside | C28H36O13 | 579.2082 | 5.099783333 | 0.095982651 |
| 79 | 4,5-Dicaffeoylquinic acid | C25H24O12 | 515.1195 | 5.136616667 | 0.093372145 |
| 80 | Glycolaldehyde dimer | C4H8O4 | 101.0243 | 0.834716667 | 0.092042109 |
| 81 | 4-Methoxycinnamic acid | C10H10O3 | 179.0705 | 6.6111 | 0.088297504 |
| 82 | Vomifoliol | C13H20O3 | 207.1382 | 5.030116667 | 0.086654213 |
| 83 | Genistin | C21H20O10 | 433.1132 | 5.030116667 | 0.086444422 |
| 84 | Astilbin | C21H22O11 | 449.1091 | 4.990216667 | 0.086207555 |
| 85 | Apigenin 5-O-glucoside | C21H20O10 | 477.104 | 4.990216667 | 0.085448038 |
| 86 | Moupinamide | C18H19NO4 | 314.1385 | 5.93145 | 0.081504305 |
| 87 | Secoisolariciresinol | C20H26O6 | 361.1657 | 5.55835 | 0.081283571 |
| 88 | Apigenin-6-C-beta-D-xylopyranosyl-8-C-alpha-L-arabinopyranoside | C25H26O13 | 533.1303 | 4.68215 | 0.080274962 |
| 89 | A-D-Glucopyranoside | C21H36O10 | 493.229 | 5.777816667 | 0.078923933 |
| 90 | Alismoxide | C15H26O2 | 221.1901 | 7.009633333 | 0.076602732 |
| 91 | Methyl 5-hydroxypyridine-2-carboxylate | C7H7NO3 | 154.0502 | 4.3193 | 0.072012586 |
| 92 | Curcumenol | C15H22O2 | 235.1693 | 9.58685 | 0.071473657 |
| 93 | Quercetin | C15H10O7 | 303.0499 | 6.161816667 | 0.069151699 |
| 94 | Grosvenorine | C33H40O19 | 739.2086 | 4.664366667 | 0.067904134 |
| 95 | Pipecolic acid | C6H11NO2 | 147.1129 | 0.762883333 | 0.06724991 |
| 96 | Daidzein | C15H10O4 | 255.065 | 5.948733333 | 0.065868513 |
| 97 | Gallic acid | C7H6O5 | 169.0144 | 1.804483333 | 0.062487794 |
| 98 | Isoferulic acid | C10H10O4 | 195.0654 | 5.118083333 | 0.060534677 |
| 99 | 2-Hydroxy-6-methoxybenzoic acid | C8H8O4 | 151.039 | 6.019483333 | 0.059563114 |
| 100 | Kouitchenside G | C27H32O15 | 595.1675 | 5.155416667 | 0.056886813 |
| 101 | Dattelic acid | C16H16O8 | 335.0775 | 4.50245 | 0.055972463 |
| 102 | (+)-Balanophonin | C20H20O6 | 357.1333 | 6.539016667 | 0.055155577 |
| 103 | Geniposidic acid | C16H22O10 | 373.114 | 3.412233333 | 0.055017636 |
| 104 | Obacunone | C26H30O7 | 455.2062 | 9.830316667 | 0.053816352 |
| 105 | Umbelliferone | C9H6O3 | 163.0391 | 5.133833333 | 0.053033304 |
| 106 | Pinoresinol | C20H22O6 | 341.1386 | 6.557716667 | 0.052981675 |
| 107 | Desoxyrhaponticin | C21H24O8 | 387.1434 | 6.05445 | 0.052432536 |
| 108 | Sinapaldehyde | C11H12O4 | 209.0809 | 5.614983333 | 0.050706887 |
| 109 | Noreugenin | C10H8O4 | 191.0351 | 5.925716667 | 0.047252427 |
| 110 | 3-Hydroxycapric acid | C10H20O3 | 187.134 | 8.1643 | 0.04625853 |
| 111 | Medicarpin | C16H14O4 | 271.0964 | 8.673816667 | 0.046247897 |
| 112 | Indigotin | C16H10N2O2 | 307.0724 | 6.348883333 | 0.0452842 |
| 113 | Methyl cinnamate | C10H10O2 | 145.0648 | 9.061433333 | 0.044281301 |
| 114 | Isofraxidin | C11H10O5 | 221.0456 | 5.227333333 | 0.044095739 |
| 115 | Salidroside | C14H20O7 | 345.1192 | 3.923816667 | 0.044055415 |
| 116 | Bayogenin-3-O-[beta-d-Galactose-(1â3)-beta-D-glucuronic acid-28-O-beta-D-glucopyranoside | C48H76O21 | 989.4952 | 6.071416667 | 0.042009209 |
| 117 | Scopolin | C16H18O9 | 399.0934 | 4.2343 | 0.037697295 |
| 118 | Padmatin | C16H14O7 | 301.0708 | 6.394066667 | 0.037689773 |
| 119 | Leucic acid | C6H12O3 | 131.0714 | 4.717816667 | 0.035456015 |
| 120 | Stachydrine | C7H13NO2 | 182.0578 | 0.90425 | 0.0352282 |
| 121 | Dihydroactinidiolide | C11H16O2 | 181.1223 | 8.195683333 | 0.034591153 |
| 122 | Scoparone | C11H10O4 | 207.0653 | 5.966683333 | 0.034421521 |
| 123 | Hedysarimcoumestan B | C16H10O6 | 297.0404 | 6.810316667 | 0.034249062 |
| 124 | [6]-Gingerol | C17H26O4 | 317.1724 | 9.04485 | 0.033916492 |
| 125 | Coniferyl alcohol | C10H12O3 | 163.0755 | 4.977266667 | 0.032852488 |
| 126 | Cheilanthifoline | C19H19NO4 | 324.1242 | 4.953083333 | 0.032473458 |
| 127 | Okanin | C15H12O6 | 287.0561 | 5.521516667 | 0.030739988 |
| 128 | Hydroxycitric acid lactone | C6H6O7 | 189.0045 | 1.13585 | 0.029458121 |
| 129 | Naringin Dihydrochalcone | C27H34O14 | 581.1872 | 5.374683333 | 0.027303804 |
| 130 | 8-Gingerol | C19H30O4 | 305.211 | 10.35826667 | 0.026543072 |
| 131 | Epicatechin | C15H14O6 | 308.113 | 4.438766667 | 0.02543274 |
| 132 | Cichoriin | C15H16O9 | 339.0724 | 3.960683333 | 0.024585769 |
| 133 | Aloe emodin | C15H10O5 | 293.0419 | 8.3357 | 0.024465482 |
| 134 | Columbin | C20H22O6 | 341.1385 | 6.91985 | 0.024464281 |
| 135 | 6-hydroxy-4-methylcoumarin | C10H8O3 | 194.0813 | 5.234783333 | 0.024385321 |
| 136 | Neoliquiritin | C21H22O9 | 417.119 | 4.8621 | 0.022828576 |
| 137 | Carglumic acid | C6H10N2O5 | 191.0662 | 0.934716667 | 0.021418317 |
| 138 | Harman | C12H10N2 | 183.0922 | 4.647216667 | 0.020016318 |
| 139 | Coclaurine | C17H19NO3 | 286.1444 | 4.285366667 | 0.019920401 |
| 140 | TMC-58B | C25H26N2O3 | 447.1923 | 8.620233333 | 0.01987774 |
| 141 | Zederone | C15H18O3 | 247.1329 | 9.8657 | 0.019865835 |
| 142 | 2,6-Dimethoxybenzoic acid | C9H10O4 | 181.0508 | 5.136616667 | 0.019853627 |
| 143 | Homovanillyl alcohol | C9H12O3 | 213.0769 | 4.4131 | 0.018496013 |
| 144 | Liquiritigenin | C15H12O4 | 255.0662 | 6.0173 | 0.01787626 |
| 145 | Rutaretin | C14H14O5 | 261.0767 | 5.41105 | 0.017370502 |
| 146 | Protosappanin A | C15H12O5 | 271.0613 | 5.099783333 | 0.017123595 |
| 147 | Arjungenin | C30H48O6 | 503.3379 | 7.87335 | 0.015051788 |
| 148 | 3-Hydroxydodecanoic acid | C12H24O3 | 215.1652 | 9.942533333 | 0.014723619 |
| 149 | Hydroxytyrosol | C8H10O3 | 153.0558 | 3.541383333 | 0.014433847 |
| 150 | Sophoricoside | C21H20O10 | 431.0984 | 5.155416667 | 0.01420602 |
| 151 | 3',4'-Dihydroxyacetophenone | C8H8O3 | 151.0402 | 4.4131 | 0.014077044 |
| 152 | 2"-O-beta-L-galactopyranosylorientin | C27H30O16 | 591.1356 | 4.431216667 | 0.01401201 |
| 153 | Carvacrol | C10H14O | 133.1014 | 6.539016667 | 0.013804665 |
| 154 | Liquiritin apioside | C26H30O13 | 595.1673 | 4.826066667 | 0.013776562 |
| 155 | Fisetin | C15H10O6 | 285.0404 | 5.485366667 | 0.013728744 |
| 156 | Alanyleucine | C9H18N2O3 | 201.1246 | 2.727883333 | 0.013396189 |
| 157 | 6-Hydroxyrubiadin | C15H10O5 | 269.0455 | 8.7298 | 0.01333073 |
| 158 | 2-Hydroxyoctanoic acid | C8H16O3 | 159.1027 | 6.662433333 | 0.013221672 |
| 159 | Trilobatin | C21H24O10 | 417.1192 | 5.539566667 | 0.012849609 |
| 160 | Byakangelicin | C17H18O7 | 315.0878 | 6.423016667 | 0.012772771 |
| 161 | Arjunolic acid | C30H48O5 | 487.3427 | 9.46305 | 0.012480352 |
| 162 | 3-O-Methylgallic acid | C8H8O5 | 183.03 | 3.83355 | 0.012221193 |
| 163 | 2-Hydroxymethylanthraquinone | C15H10O3 | 239.0704 | 7.553466667 | 0.011520346 |
| 164 | 2,6-Dihydroxyacetophenone | C8H8O3 | 151.0401 | 6.238533333 | 0.011230284 |
| 165 | Royal Jelly acid | C10H18O3 | 169.1224 | 6.002433333 | 0.010738016 |
| 166 | Hesperetin 7-O-glucoside | C22H24O11 | 447.1283 | 5.389283333 | 0.010111875 |
| 167 | Steppogenin | C15H12O6 | 271.06 | 6.019483333 | 0.009902108 |
| 168 | Diosmetin | C16H12O6 | 301.0708 | 7.064866667 | 0.009244244 |
| 169 | 3',6-Disinapoylsucrose | C34H42O19 | 753.2233 | 5.155416667 | 0.009165181 |
| 170 | Narirutin | C27H32O14 | 563.1756 | 5.012616667 | 0.009085877 |
| 171 | Propyl gallate | C10H12O5 | 195.0654 | 5.598233333 | 0.008844975 |
| 172 | Medioresil | C21H24O7 | 369.1343 | 6.441333333 | 0.008831438 |
| 173 | Picrotin | C15H18O7 | 291.0873 | 5.356433333 | 0.008708703 |
| 174 | Antiarol | C9H12O4 | 183.0664 | 5.099783333 | 0.008559764 |
| 175 | Brassylic acid | C13H24O4 | 243.16 | 8.56535 | 0.008450965 |
| 176 | 2,5-Dihydroxyacetophenone | C8H8O3 | 135.044 | 5.337633333 | 0.008051933 |
| 177 | Harmalol | C12H12N2O | 245.0932 | 4.1442 | 0.007900371 |
| 178 | 4-Nitrocatechol | C6H5NO4 | 154.0148 | 5.081066667 | 0.007873103 |
| 179 | Coumarin | C9H6O2 | 147.0441 | 6.1076 | 0.007428566 |
| 180 | Apiopaeonoside | C20H28O12 | 459.1519 | 4.44835 | 0.007273526 |
| 181 | 3,3'-Di-O-methylellagic acid | C16H10O8 | 329.0305 | 6.404766667 | 0.007177572 |
| 182 | Kauniolide | C15H18O2 | 231.138 | 11.11656667 | 0.007077062 |
| 183 | Alizarin | C14H8O4 | 239.035 | 8.146416667 | 0.006412775 |
| 184 | 2-Butenoic acid, 2-methyl-, (3aS,4S,5S,6E,10Z,11aR)-6-formyl-2,3,3a,4,5,8,9,11a-octahydro-5-hydroxy-10-(hydroxymethyl)-3-methylene-2-oxocyclodeca[b]furan-4-yl ester, (2E)- | C20H24O7 | 375.145 | 5.7423 | 0.006285794 |
| 185 | Flavonol | C15H10O3 | 283.0611 | 9.905633333 | 0.005867971 |
| 186 | Indole-3-methanamine | C9H10N2 | 291.1601 | 10.60941667 | 0.005826955 |
| 187 | Alpinetin | C16H14O4 | 271.0966 | 7.13625 | 0.005646994 |
| 188 | Isorhamnetin 3-glucoside | C22H22O12 | 459.094 | 5.136616667 | 0.005636428 |
| 189 | 2-Hydroxy-4-methoxybenzoic acid | C8H8O4 | 167.035 | 6.164466667 | 0.005293269 |
| 190 | 2-Propenoic acid, 2-methyl-2,3,3a,4,5,8,9,10,11,11a-decahydro-6,10-bis (hydroxymethyl)-3-methylene-2-oxocyc lodeca[b]furan-4-yl ester | C19H26O6 | 395.171 | 6.127533333 | 0.005259151 |
| 191 | Parthenolide | C15H20O3 | 271.1304 | 8.79795 | 0.005129044 |
| 192 | Moracin M | C14H10O4 | 287.0561 | 5.925716667 | 0.005118407 |
| 193 | Methyl (-)-shikimate | C8H12O5 | 171.0653 | 1.9184 | 0.005076256 |
| 194 | Triptonoterpene methyl ether | C20H28O2 | 301.2161 | 10.98016667 | 0.005005158 |
| 195 | Isolariciresinol | C20H24O6 | 361.1642 | 5.133833333 | 0.004965058 |
| 196 | Methyl protocatechuate | C8H8O4 | 167.0352 | 4.990216667 | 0.004769979 |
| 197 | 3-Methylquinoxaline-2-Carboxylic Acid | C10H8N2O2 | 375.109 | 4.84395 | 0.004176977 |
| 198 | 2'-Aminoacetophenone | C8H9NO | 180.0666 | 6.164466667 | 0.00413643 |
| 199 | beta-Estradiol 17-acetate | C20H26O3 | 332.2219 | 10.671 | 0.003945552 |
| 200 | Rapanone | C19H30O4 | 321.207 | 10.36825 | 0.003783485 |
| 201 | Santonin | C15H18O3 | 264.1596 | 7.352616667 | 0.003638405 |
| 202 | Isoliquiritigenin | C15H12O4 | 237.0557 | 7.49195 | 0.003430553 |
| 203 | Smyrindioloside | C20H24O10 | 405.1192 | 4.7549 | 0.003372026 |
| 204 | Cucurbitacin IIb | C30H48O7 | 519.3329 | 6.533333333 | 0.003288927 |
| 205 | Steviol | C20H30O3 | 319.2263 | 10.44615 | 0.003212737 |
| 206 | Emodin-1-O-beta-D-glucopyranoside | C21H20O10 | 431.0984 | 5.356433333 | 0.003052765 |
| 207 | 2-Hydroxyanthraquinone | C14H8O3 | 223.04 | 8.019366667 | 0.002995519 |
| 208 | Zearalenone | C18H22O5 | 317.1392 | 9.46305 | 0.002961215 |
| 209 | Dihydrotamarixetin | C16H14O7 | 299.0559 | 5.760116667 | 0.002830978 |
| 210 | 6,2'-Dihydroxyflavone | C15H10O4 | 255.0653 | 6.665983333 | 0.002812811 |
| 211 | Gnetol | C14H12O4 | 245.081 | 5.1507 | 0.002797757 |
| 212 | Veratric acid | C9H10O4 | 363.1088 | 5.227333333 | 0.002749494 |
| 213 | Moracin O | C19H18O5 | 371.1139 | 7.12345 | 0.00267663 |
| 214 | 4â²-Hydroxy-2â²-methylacetophenone | C9H10O2 | 168.1021 | 5.632283333 | 0.002668705 |
| 215 | Griffonilide | C8H8O4 | 151.0389 | 1.9184 | 0.002250081 |
| 216 | 7-Hydroxyflavanone | C15H12O3 | 285.0767 | 8.001133333 | 0.002197111 |
| 217 | 3'-O-Acetylhamaudol | C17H18O6 | 301.107 | 10.07585 | 0.002038953 |
| 218 | Tropine | C8H15NO | 142.123 | 4.752133333 | 0.001907148 |
| 219 | Neopuerarin A | C21H20O9 | 415.1033 | 4.808033333 | 0.001874556 |
| 220 | Pentamethylquercetin | C20H20O7 | 353.1029 | 8.4561 | 0.001734507 |
| 221 | gamma-Hexalactone | C6H10O2 | 227.129 | 4.898816667 | 0.001631817 |
| 222 | Salicin | C13H18O7 | 267.0875 | 3.797683333 | 0.001631589 |
| 223 | Isovanillin | C8H8O3 | 135.0443 | 4.926766667 | 0.001556571 |
| 224 | 5,7-Dihydroxycoumarin | C9H6O4 | 179.0343 | 4.769616667 | 0.001413551 |
| 225 | Hyoscyamine | C17H23NO3 | 290.1758 | 4.5605 | 0.001275506 |
| 226 | Methyl dihydrojasmonate | C13H22O3 | 227.164 | 10.09441667 | 0.001249586 |
| 227 | Ajugol | C15H24O9 | 366.1768 | 3.54675 | 0.00119074 |
| 228 | Sedanolide | C12H18O2 | 195.138 | 10.23475 | 0.001183166 |
| 229 | Zingerone | C11H14O3 | 239.0924 | 5.760116667 | 0.000911441 |
| 230 | Thyminose | C5H10O4 | 133.0506 | 0.818033333 | 0.000675375 |
