# Supplementary material for: Shen-Bai-Jie-Du decoction suppresses the progression of colorectal adenoma to carcinoma through regulating gut microbiota and short-chain fatty acids
Source: Chin Med. 2024 Oct 28;19:149. doi: 10.1186/s13020-024-01019-4 (PMC11514841; doi:10.1186/s13020-024-01019-4)
Supplement: Supplementary file 4 — Additional file 4 [file 13020_2024_1019_MOESM4_ESM.docx]

**Supplementary Table 3. Sequences of qPCR primers used in this study.**

| **Primers** | **Sequences (5’-3’)** | |
| --- | --- | --- |
| Faecalibaculum rodentium | Forward | CCGGGAATACGCTCTGGAAA |
|  | Reverse | GCCAACCAACTAATGCACCG |
| Ruminococcuss spp. | Forward | GGCGGYTRCTGGGCTTT |
|  | Reverse | CCAGGTGGATWACTTATTGTGTTAA |
| Faecalibacterium prausnitzii | Forward | GATGGCCTCGCGATTAG |
|  | Reverse | CCGAAGCCTTCTTCCTCC |
| 16S rRNA | Forward | ACTCCTACGGGAGGCAGCAGT |
|  | Reverse | ATTACCGCGGCTGCTGGC |
| *GPR41* | Forward | CTTCTTTCTTGGCAATTACTGGC |
|  | Reverse | CCGAAATGGTCAGGTTTAGCAA |
| *GPR43* | Forward | CTTGATCCTCACGGCCTACAT |
|  | Reverse | CCAGGGTCAGATTAAGCAGGAG |
| *GPR109a* | Forward | TTGGCTATGAACCGACAGGG |
|  | Reverse | CCTCGCCATTTTTGGTCATCA |
| *HDAC1* | Forward | AGTCTGTTACTACTACGACGGG |
|  | Reverse | TGAGCAGCAAATTGTGAGTCAT |
| *HDAC3* | Forward | ACCGTGGCGTATTTCTACGAC |
|  | Reverse | CAGGCGATGAGGTTTCATTGG |
| *IL-1β* | Forward | CTTCAGGCAGGCAGTATCACTC |
|  | Reverse | TGCAGTTGTCTAATGGGAACGT |
| *IL-6* | Forward | ACAACCACGGCCTTCCCTAC |
|  | Reverse | TCTCATTTCCACGATTTCCCAG |
| *IL-10* | Forward | GCTCTTACTGACTGGCATGAG |
|  | Reverse | CGCAGCTCTAGGAGCATGTG |
| *β-Actin* | Forward | GTATGCCTCGGTCGTACCA |
|  | Reverse | CTTCTGCATCCTGTCAGCAA |
